# Bio-inspired contour extraction via EM-driven deformable and rotatable directivity-probing mask

**DOI:** 10.1038/s41598-022-16040-6

**Published:** 2022-07-19

**Authors:** Jung-Hua Wang, Ren-Jie Huang, Ting-Yuan Wang

**Affiliations:** 1 Department of Electrical Engineering, National Taiwan Ocean University, Keelung City, 20224 Taiwan; 2 AI Research Center, National Taiwan Ocean University, Keelung City, 20224 Taiwan; 3 Industrial Technology Research Institute (ITRI), Hsinchu, 310401 Taiwan

**Keywords:** Electrical and electronic engineering, Computational science, Computer science, Information technology

## Abstract

This paper presents a novel bio-inspired edge-oriented approach to perceptual contour extraction. Our method does not rely on segmentation and can unsupervised learn to identify edge points that are readily grouped, without invoking any connecting mechanism, into object boundaries as perceived by human. This goal is achieved by using a dynamic mask to statistically assess the inter-edge relations and probe the principal direction that acts as an edge-grouping cue. The novelty of this work is that the mask, centered at a target pixel and driven by EM algorithm, can iteratively deform and rotate until it covers pixels that best fit the Bayesian likelihood of the binary class w.r.t a target pixel. By creating an effect of enlarging receptive field, contiguous edges of the same object can be identified while suppressing noise and textures, the resulting contour is in good agreement with gestalt laws of continuity, similarity and proximity. All theoretical derivations and parameters updates are conducted under the framework of EM-based Bayesian inference. Issues of stability and parameter uncertainty are addressed. Both qualitative and quantitative comparison with existing approaches proves the superiority of the proposed method in terms of tracking curved contours, noises/texture resilience, and detection of low-contrast contours.

## Introduction

**Figure 1:**
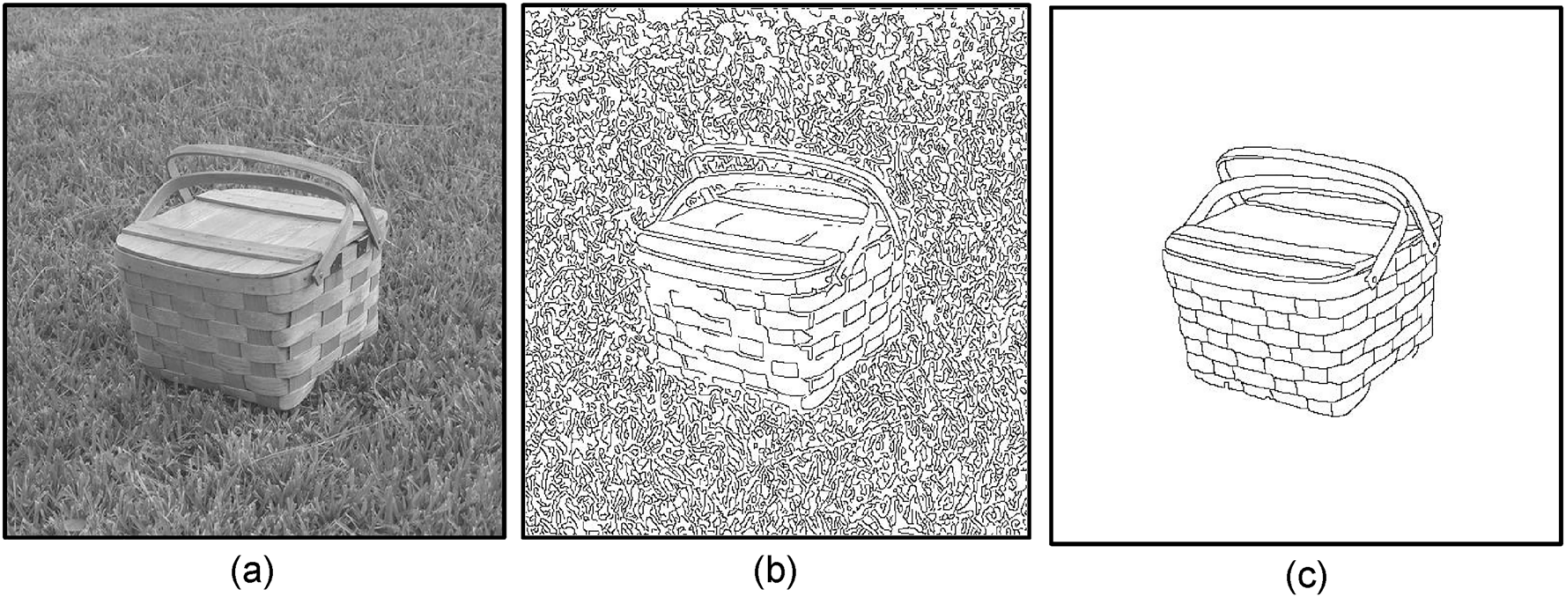
(**a**) A basket and grasses in the background. (**b**) Result of Canny detection^4^, $H_{t} = 0.2$, $L_{t} = H_{t} \times 0.4$. (**c**) Contours perceived by the human visual system.

Most early approaches to contour extraction mainly aim at quantifying the presence of boundary or tracking edge pixels at given image locations through local measurements using a fixed-shape filtering mask. The terms edge and boundary have often been used interchangeably for many years. Edge detection identifies points in a digital image at which the image intensity changes sharply or has discontinuities, it may filter out less relevant information while preserving the important structural properties of an image, allowing to reduce the amount of data to be processed significantly. Finding edges has long been regarded as a core means in various computer vision problems such as image segmentation^1^, pattern recognition^2^, and motion tracking^3^. By far the most prevalent method for edge detection is the gradient-based methods, to name a few^4,5^, the Sobel, Roberts, Prewitt operators detect edges by convolving a gray-scale image with first-order derivative filters. The Laplacian of Gaussian Operator (LoG) uses second-order derivative. The Canny detector^4^, equipped with advanced features like nonmaximal suppression (NMS) and dual thresholding, is the most recognized gradient-based edge detector that can effectively exploit the pixel intensity discontinuity. Figure 1b shows excessive redundant textures and false edges (false positives, FP) preserved by Canny detector using dual thresholds $H_{t}$ = 0.2 and $L_{t}$ = 0.08. If an edge pixel’s normalized gradient magnitude $g$(*x,y*) > $H_{t}$, it is marked as a strong edge. If $g$(*x,y*) > ${ } L_{t}$ and $g$(*x,y*) < $H_{t}$ , it is marked as a weak edge. If $g$(*x,y*) < $L_{t}$, it will be suppressed and removed. Although the Canny detector also uses an edge tracking strategy called *hysteresis*, which is basically a process of Connected Components Labeling, to remove some unqualified weak edges (i.e. false positives) possibly caused by noise. However, when it comes to more complex nature images, different local image areas may require rather different threshold values to achieve satisfactory results. The two threshold values are empirically determined and their definition will depend on the content of a given input image. All the aforesaid gradient-based methods either suffer from noises and textural interferences, or may require tedious work of empirically tuning parameters to fit different image content natures. Particularly, the sensitivity to noise and clutter in these detectors often yields false edges or fragmented details, which are undesired in cases where only contours of semantic objects as perceived by the human mind^6^ are needed. Take Fig. 1 as an example, the human visual system tends to focus only on the picnic basket in Fig. 1c, while neglecting cluttering objects such as the grasses.

Although contours are often obtained from edges, in practice they are aimed at being object contours*.* Contour extraction is seen as a distinctively different task from edge detection, it aims to find the boundaries between what humans would consider to be semantically different objects or regions of the image. Traditional contour extraction approaches are roughly divided into three categories: gradient-oriented, edge-oriented, and region-oriented. Among them, the edge-oriented approach is considered having the most balanced performance between detection accuracy and computational complexity. Many edge-oriented methods^7,8^ implement contour extraction as a two-stage process: edge detection followed by edge grouping. Ideally, a set of edges can be grouped, after the edge detection, for defining object boundaries. Unfortunately, traditional solutions for edge grouping often require a global optimization, whereby information from the entire image is taken into consideration simultaneously. For example, the work of^8^ combines multiple local cues into a globalization framework based on spectral clusters and calculates the eigenvectors thereof, with the eigenvectors themselves carrying contour information. The difficulty of which is that the optimization problem is *NP*-hard, making it unlikely to find the solution in a reasonable time.

To avoid the *NP*-hard difficulty, a nice alternative to edge grouping strategy is the gestalt laws. The word gestalt is German for “unified whole”. Since the 1920s, many gestalt psychologists have identified a set of gestalt laws^9^ helpful in accounting for the observation of how complex scenes can be reduced to more simple shapes by humans. Because the gestalt laws are the factors that lead to human visual perception, they naturally have been used as guidelines for *grouping* edges. In^10–12^, they pointed out that textured areas often exhibit stronger local luminance changes than object contours and conjectured that the *low-level* edge features alone cannot be reliable indicators of the presence of a contour. gestalt laws were incorporated into their design of a morphological operator to exploit higher-level features. Three of these gestalt laws are most related to edges grouping: Proximity, Similarity, and Continuity. The similarity law says that elements that are similar, in attributes of color, size, shape, and orientation, are easier to be perceived as a unified group than dissimilar elements. The proximity law states that things that are close together appear to be more related than those spaced farther apart. In some cases, proximity is so powerful that it can override the similarity of color, shape, and other factors that might differentiate a group of objects. The law of continuity states that people tend to perceive objects in alignment as forming a *smoothest* line or curve.

More recently, deep learning (DL) models has been shown effective^13–15^ in extracting object contours for various open datasets. DL is a new branch of machine learning (ML) and was introduced by Hinton et al.^16^. It is nowadays considered as one of the hot topics in the context of computing, especially in the field of computer vision. However, building an appropriate DL model is a challenging task, due to the dynamic nature and variations of real-world problems and data. Moreover, interpretability is an important factor when comparing DL with traditional ML algorithms. So far, it is still very difficult to explain how a deep learning result was obtained. DL models are typically considered as “black-box” machines that hamper the standard development of research and applications. Performance of DL models must rely on *accurate and consistent* labeled data, meaning intensive labor workload is needed for data labeling and cleaning, let alone the issue of *labeling bias* that may arise, particularly when labeling *subjective data* by plural labelers with differences in experience, preference, and understanding of the assignment. Besides, in order to avoid the overfitting problem, huge amount of training data is required. In some cases, the amount of training data may never be satisfied! This is particularly true in applications involved with nature scene images, for example, pretrained DL models built on a self-driving car is very likely to see a scene that was too different from what it had been trained on it. Although car makers could always constantly update its DL models to deal with corner cases, the problem is, these corner cases are virtually limitless, which is often referred to as the “long tail” problem of DL.

Nevertheless, some interesting contour detection results generated by DL have been reported. HED (Holistically-Nested Edge Detection)^13^ and RCF (Richer Convolutional Features)^14^ are two highly cited works, both focusing on image-to-image transformation (i.e. coder-decoder) for yielding a coded feature vector for an input image. They are essentially region-oriented extractors built on a convolutional neural network (CNN) paradigm. Currently the most popular strategy for training CNN, however, is the backpropagation algorithm^17^, which is well known for its slow training speed. Also, such “black-box” design may not be appropriate for some applications (e.g., medical image diagnostics) where interpretability is highly requested, let alone the pixel-level accuracy required to pinpoint the exact location and area of a lesion. Clearly, accurate edge positioning is needed, and so are the in-depth investigations on low-level characteristics of real image edges.

The human visual system can quickly digest complicated scenes into simple meaningful object contours. It is commonly acknowledged that the human visual system operates by performing low-level task of detecting light intensity discontinuities (edges) followed by mid-level, and higher levels semantic representation of object-like structures. All the information required to build the high-level semantic representation must already represented as mid-level features such as the orientations of edges that characterize an object contour. Little is known about the mid-level process, except some knowledge about the size of receptive field or sensory space become larger from the low-level of retinal ganglion cells and LGN (lateral geniculate nucleus) to the mid-level of the visual cortex. Recently, a research area concerning computational theory of visual receptive fields has increasingly received attentions^33^. It deals with building idealized models similar to the biological receptive fields found in the retina ganglion cells, LGN, and the visual cortex, as well as derivations of theoretical explanation of the computational function of visual receptive fields. Certainly, consistent representation of image structures over multiple spatial and temporal scales are required for possible applications in computer vision. These observations make us believe that a mid-level vision task of detecting *perceptual contours* (e.g. salient boundaries of the picnic basket in Fig. 1c) is worth studying. Such mid-level vision task not only benefits by deepening our knowledge of the low-level feature extraction but also facilitates future high-level tasks such as object detection^18^, object proposal generation^19^, and sketch-based image retrieval (SBIR)^20,21^. Embodiments of these applications rely on using quality contours (not necessarily closed contours) as input data for the subsequent tasks of image segmentation or recognition etc., because if there are too many false or fragmented edges, they could greatly hamper the overall performance.

There exists a need to develop a novel approach capable of extracting perceptual contours without the problems encountered in aforesaid approaches, namely, false/fragmented edges, tedious parameter tuning, NP-hard global optimization, laborious data labeling, and low interpretability. To achieve this goal, a special sampling mask is designed to iteratively rotate and deform in search of a *principal direction* that characterizes the existence of perceptual contours. Upon convergence, Bayesian theory is employed to determine whether the target pixel is located on a perceptual contour. The deformability/rotatability property, which is generated by an unsupervised EM-algorithm, desirably *enlarges the effective size of receptive field* for exploiting Shannon probabilistic model of local image regularities. Such a unique design allows not only to utilize the low-level features that is vital to the successful detection of a single edge point, but also to spatially exploit mid-level features that are essential to extract curved contours. Mathematically rigorous derivations for the proposed EM-based Bayesian framework are provided to facilitate objective qualitative analysis and quantitative evaluation.

The rest of the paper is organized as follows. Research work related to this paper is highlighted, we review the current limitations and the need for new methodology on perceptual contours extraction. Next, we stage by stage elaborate our method. In particular, the theory underlying the proposed deformable/rotatable directivity-probing mask, which can deform and rotate to probe the principal direction over an image region (receptive field), is explained with three Hypotheses of human perception and concepts. Following that, extensive empirical results including comparisons with mainstream prior arts are provided. Finally, concluding remarks and possible future improvements are given.

## Related work

Numerous researchers in the past two decades have attempted to detect perceptual contours in various ways. Various local methods emerged addressing the issue of efficiency. LSD (Line Segment Detection)^22^ was one of the first approaches to achieve fairly good results using local features. Taking a further step^7,10,11^, considered *gestalt laws* for edges grouping in performing the contour depiction. Specifically, Elder and Goldberg^7^ employed the inferential power of gestalt laws of proximity, continuity, and luminance similarity. They found that these laws are approximately uncorrelated, suggesting a simple factorial model for statistical inference. Neurophysiological evidence shows that a non-classical receptive field (NCRF) possesses a mechanism, called surround suppression, which inhibits the response of a contour edge in the presence of other similar features in the surroundings. To simulate NCRF, Grigorescu^10^ introduced an inhibition term, which is supposedly high on textures and low on isolated edges, to the Canny detector and a Gabor function^5^. While such an approach seemingly leads to better discrimination between object contours and texture edges than methods solely based on the gradient magnitude, it has two drawbacks: first, a phenomenon called *self-inhibition* occurs, i.e., neighboring pixels and the contour edge itself inhibit each other so that the inhibition term is quite high on isolated contours too; second, a parameter called “inhibition level” needs to be introduced, whose value is left to heuristics. Papari^11^ further enhances the texture suppression effect by splitting the surround inhibition. However, as noted in^23^ the effectiveness of reducing self-inhibition hinges on the assumption there are few textures and meaningless edges around the object contour edge. In reality, most natural images usually have many meaningless structures surrounding a target object. Another work based on the same assumption, aiming to improve LSD, is the Edge Drawing (ED)^12^. ED works in two steps: (1) computing a set of anchor pixels, and (2) fitting the desired line to connect these anchor pixels with a greedy procedure. Despite its real-time operation, because ED performs the edge detection step by applying steps of gradient estimation and non-maximum suppression, it is susceptible to noise and hence excessive number of fragmented or false contours.

Other works related to using local statistical gradient estimation^24^, overcome challenging problems such as line scratch detection in old films. By exploiting first- or second-order derivative features^25,26^, studied the mid-level representation of edges for generalized boundaries detection. Despite these great strides, the produced edges often remain noisy or excessively fragmented and therefore cannot be directly used in higher-level applications. A probabilistic method proposed by^27^ is mainly based on gathering gradient orientations to efficiently depict aligned segments in an input image. However, only straight lines are preserved, making the reconstruction of curved contours virtually impossible. As another trial of mid-level representation^28^, has suggested *Shannon entropy* is more resilient to noises than the previous approaches based on derivatives evaluation of pixel intensity. Still, using Shannon entropy alone cannot respond to intensity patterns over the spatial domain, because it only involves the overall pixel intensity statistics collected over a fixed receptive filed.

**Figure 2:**
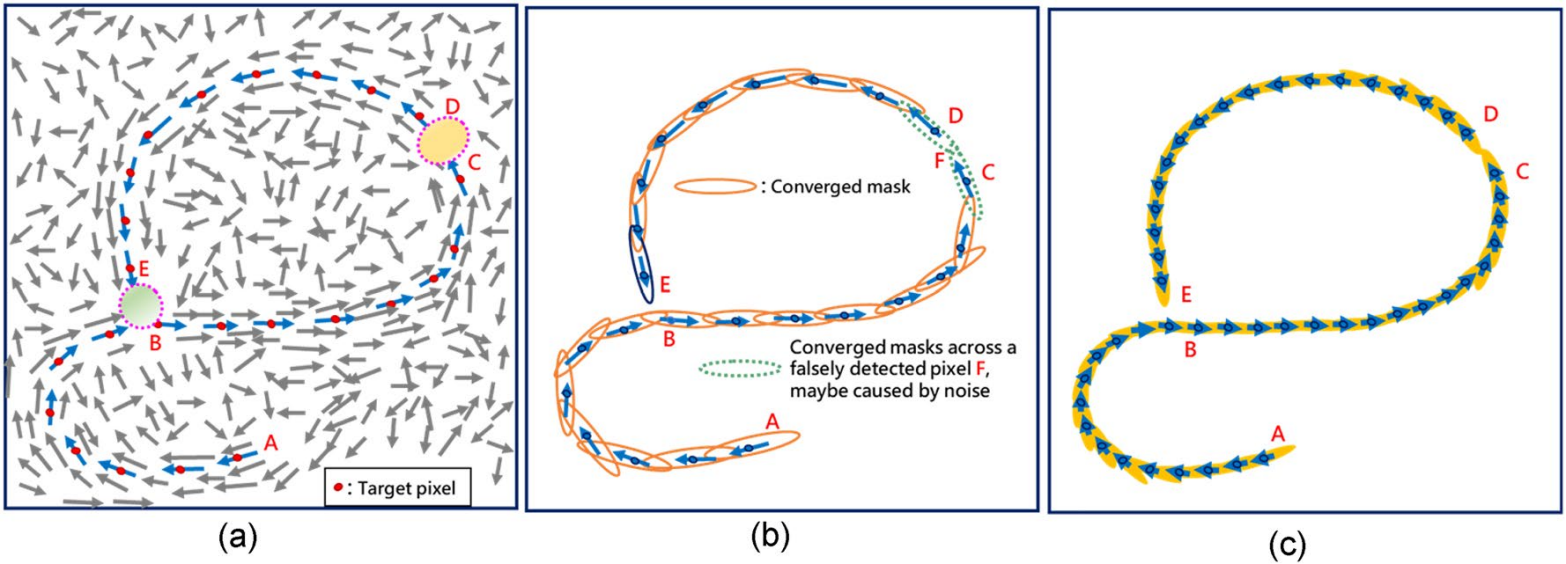
(**a**) Principal directions for a set of edges probed by the proposed deformable mask. (**b**) a set of converged masks (**c**) the corresponding contiguous contour from edge point A to edge point E.

None of the prior efforts is successful in tracking curved contours without incurring excessive fragments, textures, and false contours. Furthermore, although the ability to infer psychologically the low-level features^29^ is vital to the success of straight edge detection, they are quite susceptible to noise and interferences. Abundant studies^30–32^ of human visual system have indicated that information characterizing how successive edge pixels respond to intensity patterns (e.g. the contour in Fig. 2a) over the spatial domain must be exploited at the pixel level and beyond to tackle the task of extracting contours, especially the curved perceptual contours.

In this study an EM-driven mask is designed, in the context of computational theory of idealized receptive fields, to iteratively deform/rotate in search of the principal directions that can be used as grouping cues for contour detection. Upon convergence, the likelihood probability of a target pixel being a gestalt edge can be evaluated to allow Bayesian decision on whether the target pixel is located on a perceptual contour. Theoretical analysis of the likelihoods is provided to justify the invoking of Bayesian inference to deduce the posterior probability for the target edge pixel. Decision based on the converged likelihoods is proved to coincide with the optimality requirement in Bayes rule. All theoretical derivations, qualitative analysis, and parameters updates are conducted under the framework of EM-based Bayesian inference. The only simple assumption made here is that edges defining the boundary of perceivable objects should spatially align with a *principal direction* found by the converged mask. Accordingly, there is no need for the high complexity of choosing the right boundary gap measures from a sea of contour fragments as in^7,8^, and no tedious parametric setting is required.

**Figure 3:**
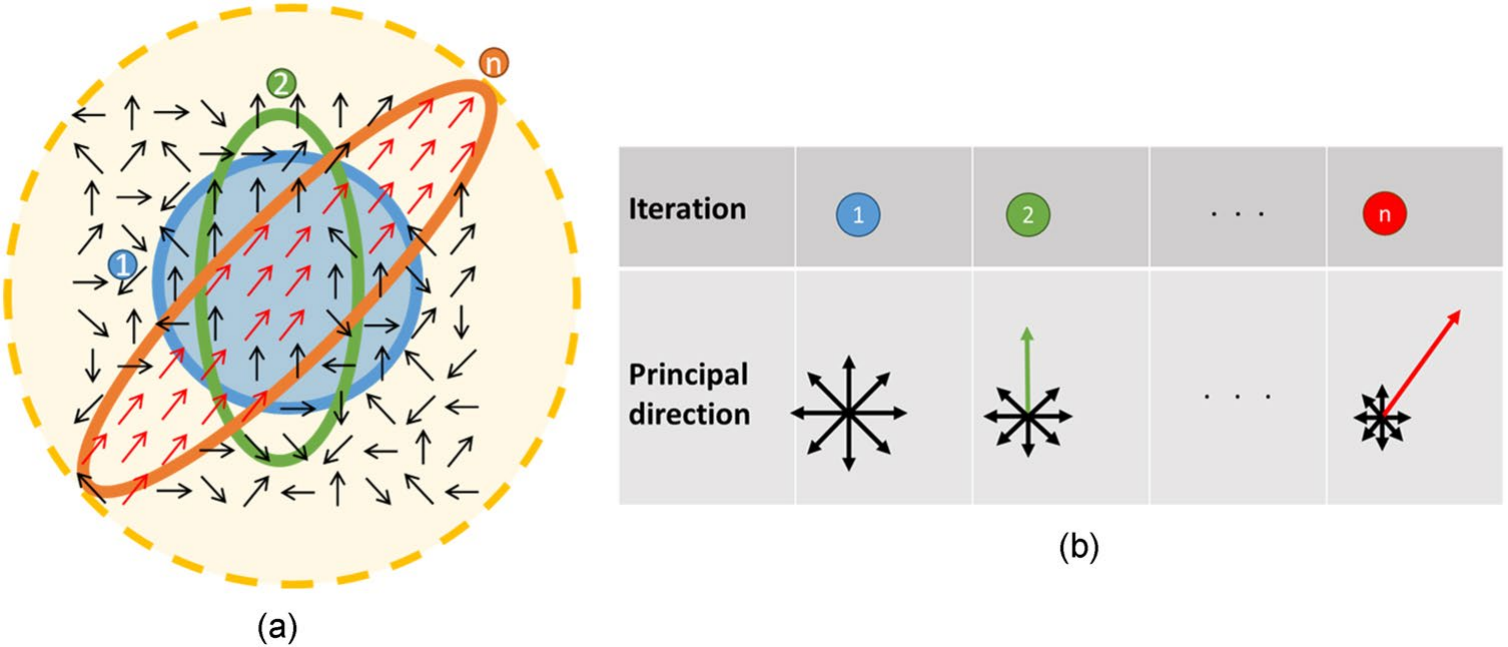
(**a**) Receptive field effective enlarged after a repetitive sequence of deform-and-rotate. (**b**) The principal direction found for (**a**) upon convergence at $$n^{th} $$ iteration.

Our method uses solely orientation data for training the EM algorithm, the deformable/rotatable mask enables the unsupervised learning of the Shannon probabilistic model of the local directional regularities. As schematically shown in Fig. 2, the convergent masks (each centered at a gestalt edge, red dot) would be aligned with the principal direction of gradients (blue arrows in Fig. 2b), and together they could track in relays the contiguous edges of the same contour (Fig. 2c) while suppressing nearby noise and textures. With our method, the edge points E and B are too distinctly different in their principal directions, their convergent masks do not overlap, they will not be grouped into the same contour. Despite edge points C and D are apart from each other due to a falsely detected pixel therebetween, they share high similarity in the principal direction, and may afterwards be connected into the same contour. Unlike the NCRF in^10^ and the splitting surround inhibition in^11^, our mask provides an ideal receptive field that is free of the self-inhibition problem and no assumption of few textures/meaningless edges around the object contour edge is needed. Moreover, as will be seen in Fig. 3, the EM-driven scheme can enlarge the effective size of receptive field, making it especially suitable for extracting perceptual contours by characterizing how edge pixels respond to intensity patterns over the spatial domain. The deformation/rotation not only facilitates pixels having the same or similar orientations to be covered by the mask, but also help discriminate a target pixel from pixels that have dissimilar gradient orientations, ensuring resilience to noises and interferences^28^. In contrast to the prior works on perceptual contours, this study provides a unified approach that outperforms in tracking curved contours (vs. gestalt-based methods^7,10,11,28^), less susceptible to noises (vs. gradient-based methods^12,24–27^) more interpretable and mathematically rigorous (vs. deep learning methods^13–15^), lower parameters uncertainty (vs. all prior arts).

The overall contribution of this paper is summarized as follows:

- Realization of a novel contour extractor capable of producing results in good agreement with gestalt laws, without having to perform *NP*-hard global optimization for an edge-connecting mechanism.
- Introducing a deformable/rotatable sampling mask driven by an unsupervised EM algorithm to create an ideal receptive field, which is free of surround suppression and self-inhibition problems.
- Casting the problem of contour detection as a local optimization problem, all theoretical derivations are conducted under the framework of EM-based Bayesian inference.
- Physically quantifying two metrics of *principal direction* and *belief* thereof, and showing their usage as an effective grouping cue for contour detection.
- Using solely orientation data for training the EM algorithm enables the convergent masks to track, in relays, the contiguous edges of the same contour while suppressing noise and textures.

## Methods

### Hypotheses made about human vision

Because many neuroscience and other cognitive sciences provide merely evidence, laws and explanations of the functioning principles of certain aspects of visual perception in the human brain, to benefit from these principles and laws in their implementations on a computer vision task, it is necessary to carefully investigate the suitability of theoretical elements before taking them into actual design considerations of methodology. In the following discussions, we elaborate on three hypotheses of human perception that best fit our needs set forth in the previous sections. One hypothesis regards the biological evidence of receptive fields of cells in the visual cortex, their larger size preference and the sensitivity to image orientations have inspired us to devise a deformable mask for probing the principal direction over an *enlargeable* image region. The other two hypotheses have strong connections with the gestalt laws.

Hypothesis-1 is based on the historical finding of Hubel^32^ regarding the receptive fields in the visual cortex are larger and images for these receptive fields need to have an orientation to excite the cell. They can be tuned to different sizes, orientations and even motion directions in the image domain, enabling the visual system to compute invariant image representations at higher levels in the visual hierarchy. Inspired by Hypothesis-1, numerous methods^34,35^ employed a *fixed* large sampling mask to determine principal orientations of pixels, yet they failed to preserve smoothly varying contours. In this study, we introduce an idealized receptive field, which is not fixed in its shape and size and capable of imitating the ability of the human visual cortex to perceive the picnic basket in Fig. 1c easily while ignoring the grasses on the ground. Hypothesis-2^29^ states that sensory representations are adapted to the *local statistics* in sensory signals. In the human visual system, sensory signals may simply refer to physical illumination and changes of which would cause significant changes in neuronal activity. Luminance gradients should encode essential information of a given object at early stages in the visual system. Humans can even convert the gradient of neighboring pixels with similar gradient orientations to illusory representations. For instance, the Craik-O’Brien-Cornsweet illusion and Müller-Layer illusion^36^. Furthermore, it has been found^30^ that gradients at object contour arguably provide the most fundamental cues to our visual system for identifying the edge location of object. Hypothesis-3^32^ states that the human visual system also perceives orientation alignment continuity at the pixel level, implying a mechanism different from that of region-based edge detectors must be employed by the human visual system to allow the perception of smoothly varying contours.

To understand our design concept, it helps to elaborate on the connection between our method and the above hypotheses. Firstly, based on Hypothesis-2, we conjecture that the ability to statistically compile gradients must be functionally related to the process involved in detecting continuous edges. In this regard, we thus incorporate the gestalt laws of similarity and proximity, which explicitly point out that the *resemblance* in gradient orientations of *proximal* pixels plays an important role in visual perception, into the computational design of the mask operation. According to Hypothesis-2, the closer a target pixel is located to its neighboring pixels with similar gradient directions, the more likely they together form a set of contiguous edges to become perceivable or even illusory patterns. Considering that the gradient vector always points in the direction of the greatest intensity change, and the length of it corresponds to the rate of change in that direction, we will use a gradient operator to calculate entropy and related probabilistic measures. Furthermore, since image entropy is an inverse indicator of direction uniformity, the gradient histogram of sampling pixels is adopted in this work to evaluate the entropy and hence likelihoods of the two classes $\Omega$ and $\overline{\Omega }$. This is motivated by the intuition that contours correspond to image discontinuities, and histograms provide a robust mechanism for modeling the content of an image region. Secondly, since a set of neighboring pixels having similar gradient angles might constitute a meaningful contour, presumably many such sets nearby should together form a spatially varying pattern over a large receptive field. This, along with Hypothesis-1, inspire us to propose a deformable/rotatable sampling mask. Compared to a static mask, such a dynamic mask should be much more effective in finding the pertaining sets of pixels because it has a better chance of visiting pixels that are otherwise unreachable, thus ideally enlarging the effective size of receptive field using a single sampling mask. Lastly, our method evaluates a pair of likelihoods for a set of candidate edge pixels (CEP) generated by, in principle, any general edge detectors such as Canny detector, Gabor wavelet^4^, Sobel operator, etc., or even the combination thereof. In processing a candidate pixel, the mask iteratively deforms and rotates in search of the existence of a principal direction that characterizes that most important mid-level feature associated with the candidate pixel. We will show that such a direction-aware mask evaluates not only the overall orientation alignment among the currently sampled pixels, but also the relation between the target and its neighborhoods.

### Enlarging the effective receptive field

The deformable/rotatable sampling mask really lies at the heart of our localization machinery, as it enables more effective probing of the principal direction by enlarging the receptive field. To see this, we use the schematic diagram in Fig. 3 where an exemplar image region containing 100 arrows is shown, with each arrow representing a pixel gradient direction (i.e., the level-line field in^22^). Many red arrows at the diagonal area indicate the existence of an image contour having a principal direction of 45°. The question is how to effectively and stably locate these pixels and identify the principal direction (i.e., the red line in Fig. 3b). Using the center point in Fig. 3a as the target point, we start with a circular sampling mask. Clearly, arrows covered by that mask mostly are 90°. As will be derived later, through calculating the likelihood of the class (gestalt or non-gestalt) w.r.t the target point, the sampling mask will deform in response to that majority angles of 90° at 2nd iteration to become somewhat vertically elongated. Iterating in this way and letting the mask deform and align its long axis with the updated principal direction of sampled pixels, the mask will eventually stop deforming. Upon convergence in the *n*th iteration, the converged long axis of the mask coincides with the principal direction of 45°. Significantly, due to the elongation and rotation, the actual sampled area (noted by the dashed yellow circle) is much greater than that of the initial sampling mask. A larger receptive field allows capturing more spatial context, thus increasing the ability to detect larger and more complex spatial patterns while neglecting smaller fragmented edges caused by noises. Entropy calculation within the converged mask allows us to assess, in addition to the overall gradient statistics, the similarity degree of a target pixel w.r.t its neighboring pixels. The deformation is driven by an EM algorithm especially tailored for adjusting the shape of the mask, and it can desirably respond to perceivable patterns and maximize the expectation likelihood w.r.t the target pixel.

In this work, a pair of probabilistic measures ∈[0,1] is defined as the *latent* variables in the EM algorithm. We will formally show how the EM algorithm is trained to iteratively adjust these two measures, which in turn control the deformation and rotation of the mask. Upon convergence, the mask optimally aligns with the principal direction of the sampled pixels. Also, with the proper arrangement of training data to be detailed below, the two measures can serve as the likelihoods for gestalt edges and non-gestalt edges, respectively. In the context of Bayesian inference, this allows invoking the Bayes rule to determine whether the target pixel is located on an object contour as perceived by humans.

Our method mainly comprises the following steps: First, to generate two feature maps and a set of CEP; picking an unprocessed edge pixel from CEP as a target point and subjecting to the EM algorithm to iteratively update, using the feature maps as training data, the shape and rotation angle of the sampling mask. Upon convergence, invoking the Bayes rule^37^ to determine whether the target pixel is a gestalt edge, if not, it will be removed; subjecting the next unprocessed pixel to the EM algorithm until all elements in CEP are processed; finally, the remaining (preserved) pixels are outputted as the extracted contours.

### Training data for EM algorithm

As in other machine learning algorithms, the EM algorithm requires (unlabeled) training data. In this work, two feature maps are generated from the input image, they provide the basic low-level features for learning mid-level representation by the EM algorithm. To prepare the training data, we may optionally apply a prefilter of low-pass Gaussian^38,39^ or median filter to a raw input image to obtain an output image *I*_*mg*_. Following that, gradients $g_{x}$ and $g_{y}$ are obtained by applying any gradient operators^4^ such as Sobel, Roberts or Prwitt to *I*_*mg*_. We then use the formulas of ${ }\sqrt {g_{x}^{2} + g_{y}^{2} }$ and $\arctan \left( {g_{x} /g_{y} } \right)$ to obtain a magnitude matrix ${\varvec{g}}$ and an orientation matrix ${\varvec{\theta}}$, respectively. Afterward, a normalized $g^{N} \left( {x,y} \right)$ is obtained by setting the upper and lower extreme elements in $\log \left( {{\varvec{g}}^{N} } \right)$ to 1 and 0, respectively. Elements in ${\varvec{\theta}}{ }$ are real numbers ∈ [− 90°, 90°].

In preparing the feature map ${\varvec{\theta}}_{\tau} { }$ in association with class $\Omega$, for any pixel $\left( {x,y} \right)$ with $g^{N} \left( {x,y} \right) \le\tau$, $0 < \tau< 1$, we replace its original $\theta \left( {x,y} \right)$ with a null value, indicating it will not take part in the histogram computation later on. Based on our extensive experiments (not shown here), it is found that satisfactory performance can generally be obtained by setting the parameter *τ* to median or mean of elements in the quantized matrix ${\varvec{\theta}}$, which is not surprisingly in line with our intuition. Analogous to NMS (Non-maximum suppression) used in Canny detector, such a null-value assignment helps reduce noises and neglect insignificant intensity changes. Likewise, in preparing the feature map $\overline{{{ }{\varvec{\theta}}}}_{\tau}^{} { }$ in association with class $\overline{\Omega }$, for any pixel with $g^{N} \left( {x,y} \right) >\tau$, we replace its original $\theta \left( {x,y} \right)$ with a null value. Now, ${\varvec{\theta}}_{\tau}$ should consist of two subsets ${\varvec{\theta}}_{ \tau}^{1} {\text{ and }}{\varvec{\theta}}_{ \tau}^{2}$ that contain orientation data with $g^{N} \left( {x,y} \right) >\tau$ and $g^{N} \left( {x,y} \right) \le\tau$, respectively. Because $n\left( {{\varvec{\theta}}_{ \tau}^{1} } \right) + n\left( {{\varvec{\theta}}_{ \tau}^{2} } \right)$ equals the image size, the map ${\varvec{\theta}}_{\tau}$ carries all the information needed for evaluating the likelihood $p\left( {x,y|\Omega } \right)$. Similarly, $\overline{{\theta }}_{\tau}^{}$ consists of two subsets ${ }\overline{{\theta }}_{\tau}^{1} {\text{ and }}\overline{{\theta }}_{\tau}^{2}$ that contain orientation data for pixels with $g^{N} \left( {x,y} \right) \le\tau$ and $g^{N} \left( {x,y} \right) >\tau$, respectively. The map $\overline{{\theta }}_{\tau}^{}$ carries all the information needed for evaluating the likelihood ${ }p\left( {\overline{\Omega }|x,y} \right)$. The likelihood $p\left( {x,y|\Omega } \right)$ of $\Omega$ w.r.t the target pixel $\left( {x,y} \right)$ is a term chosen to indicate that the class $\Omega$, for which $p\left( {x,y|\Omega } \right){ }$ is large, is more “likely” to be the true class. Our goal is to calculate the posterior ${ }p\left( {\Omega x,y} \right)$ using the information evaluated by the EM algorithm. Note that $p\left( {\Omega x,y} \right) + p\left( {\overline{\Omega }|x,y} \right) = 1.$ Shortly we will see that in implementing the EM algorithm, the maps ${\varvec{\theta}}_{\tau} {\text{ and }}\overline{{ {\varvec{\theta}}}}_{\tau}^{}$ can be conveniently used as the probability distributions governing the latent variables $p\left( {x,y|\Omega } \right)$ and $p\left( {x,y|\overline{\Omega }} \right)$, respectively. For simplicity, we may use symbols $p{ }$ and $\overline{p}$ to denote $p\left( {\Omega |x,y} \right)$ and $p\left( {\overline{\Omega }|x,y} \right)$, respectively.

### Generating CEP

Considering that gestalt edges must be edges, yet the converse is not necessarily true, it would be more computationally efficient to start with a set of CEP pre-identified as edge points, and then screen off those non-gestalt edges. As said, one can choose any low-level edge detectors to obtain CEP. However, due to its capability of removing spurious responses while preserving weak edges, Canny detector is ideal for use as the demonstrative generator of CEP. Besides, we can computationally benefit from the standard Canny detector, as its algorithm involves the step of generating gradients information. Unless otherwise specified, the Matlab-version Canny detector with default settings of dual thresholds ($H_{t} = 0.2$, $L_{t} = H_{t} \times 0.4$) and the smoothing parameter $\sigma = \sqrt 2$ were used.

### Evaluating likelihoods under the framework of EM algorithm

One key attribute of this work is to cast the implementation of the deformable sampling mask into the framework of an EM algorithm^40^, especially the mask is driven by the EM algorithm to deform and rotate for probing the existence of a principal direction. The EM algorithm is really at the bottom of the many unsupervised algorithms in the field of machine learning. It is often used as an iterative method for solving chicken-and-egg problems by finding out the maximum likelihood or maximum a posteriori (MAP) estimates of unknown parameters in statistical models. These models depend on unobserved latent variables, i.e., variables not directly observable yet can be inferred from the observable data. The algorithm alternates between an expectation phase (E-phase) and a maximization phase (M-phase) until convergence. The E-step estimates the expected values of the hidden variables given the current values of the shape parameters and the observed data. The M-step then uses these expected values to find the set of parameters that maximize the likelihood of the data.

**Figure 4:**
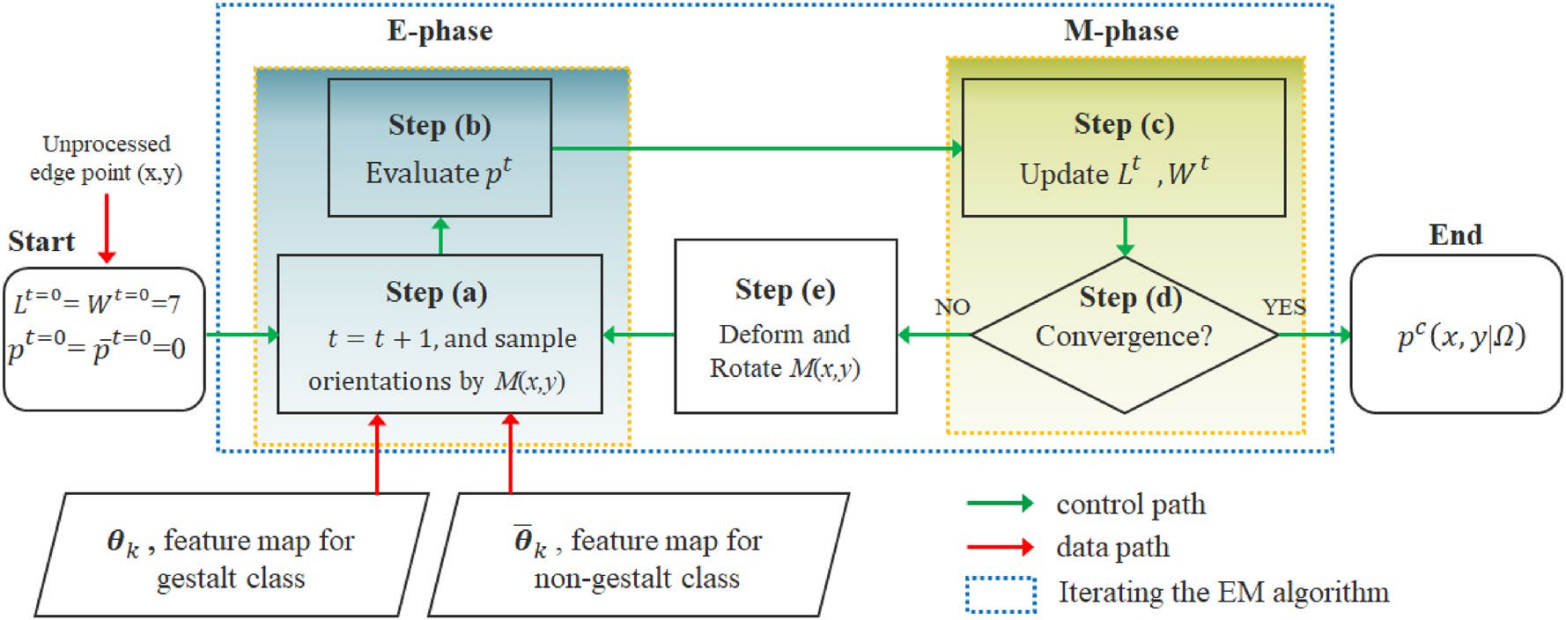
Flowchart of computing likelihood values for a target edge point.

In this work, likelihoods $p{ }$ and $\overline{p}$ are treated as latent variables, they can be evaluated from the observable data ${\varvec{\theta}}_{\tau} {\text{ and }}\overline{{{ }{\varvec{\theta}}}}_{\tau}^{}$. The flowchart of evaluating likelihood values for a target pixel is shown in Fig. 4, where the value of the long axis $L$ and the short axis $W$ of a sampling mask are treated as the unknown parameters. Note that in principle the shape of the sampling mask is not limited, just like the structure element in morphological operations^4^. Here we only use a rectangular mask for explanation purpose. Other shapes such as ellipse will generally work fine, and derivations of parameters are alike. One just needs to replace length and width with semi-major and semi-minor axes in case of using an elliptical mask. As will be proved later, a well-defined objective function will converge to maximize the likelihood $s$. The E-phase creates a function for the expectation of the likelihood values using the present estimate for the parameters *L* and *W*, whereas the M-phase computes new values of $L$ and $W$ that maximize the expected likelihood evaluated on the E-phase.

*Initialization:* Define an initial mask ${\varvec{M}}^{t = 0}$ centered at a target pixel $\left( {x,y} \right)$ picked from CEP. For ordinary image size smaller than 1024*1024, ${ }L^{t = 0}$ and ${ }W^{t = 0}$ are set to 7. The initial mask size should properly increase with the Image resolution greater than 1024. Also, denote *Bn﻿* as the total number of bins, set $p^{t = 0} = \overline{p}^{t = 0} = 0,$  $Bn=L\times W$. There is another mask ${ }\overline{{M}}^{t}$ for sampling $\overline{{{ }{\varvec{\theta}}}}_{\tau}^{}$, yet we will only focus on elaborations of ${\varvec{M}}^{t}$ for brevity.

*Step(a)*: Set *t* = *t* + *1*. refer to Fig. 4, elements in ${\varvec{\theta}}_{\tau} {\text{ and }}\overline{{{ }{\varvec{\theta}}}}_{\tau}^{}$ sampled by the mask are the observable data useful for constructing a histogram of gradient orientation, wherein $h_{i}^{t} \left( {x,y} \right)$ and $\overline{h}_{i}^{t} \left( {x,y} \right)$ represent the $i^{th}$ bin height for the sampled elements in ${\varvec{\theta}}_{\tau} {\text{ and }}\overline{{{ }{\varvec{\theta}}}}_{\tau}^{}$, respectively. Also, denote $h_{T}^{t} \left( {x,y} \right){\text{ and}}$ $\overline{h}_{T}^{t} \left( {x,y} \right){ }$ as the bins associated with the target pixel in ${\varvec{\theta}}_{\tau} {\text{ and }}\overline{{{ }{\varvec{\theta}}}}_{\tau}^{} ,$ respectively. In particular, $h_{max}^{t} \left( {x,y} \right)$ and $\overline{h}_{min}^{t} \left( {x,y} \right)$ are the highest and lowest bins among all bins sampled by the mask. Step(a) in conjunction with Step(b) correspond to the E-phase of EM algorithm.

*Step(b):* Update the likelihood $p{ }$ and $\overline{p}{ }$ using Eqs. (1a) and (1b), respectively

1a $$p^{t} = \alpha \times \left[ {\frac{{{\mathcal{H}}_{max}^{Bn} - {\mathcal{H}}^{t} \left( {x,y} \right)}}{{{\mathcal{H}}_{max}^{Bn} }}} \right],{ }$$

1b $$\overline{p}^{t} = \beta \times \left[ {\frac{{{\overline{\mathcal{H}}}^{t} \left( {x,y} \right)}}{{{\mathcal{H}}_{max}^{Bn} }}} \right],$$

2a $${\mathcal{H}}^{t} \left( {x,y} \right) = - \mathop \sum \limits_{i = 1}^{Bn} h_{i}^{t} \left( {x,y} \right) \times log\left( {h_{i}^{t} \left( {x,y} \right)} \right),$$

2b $${\overline{\mathcal{H}}}^{t} \left( {x,y} \right) = - \mathop \sum \limits_{i = 1}^{Bn} \overline{h}_{i}^{t} \left( {x,y} \right) \times log\left( {\overline{h}_{i}^{t} \left( {x,y} \right)} \right),$$

3 $${\mathcal{H}}_{max}^{Bn} = - \log \left( \frac{1}{Bn} \right),$$

where the second term on the RHS of Eq. (1a) and (1b) is defined as non-uniformity or directivity. A larger value of $\left[ {{\mathcal{H}}_{max}^{Bn} - {\mathcal{H}}^{t} \left( {x,y} \right)} \right]$ implies a higher chance of having a peak in the gradient histogram, i.e., a principal direction. The parameter $\alpha$ is defined as ${ }h_{T}^{t} \left( {x,y} \right)/h_{max}^{t} \left( {x,y} \right),$ it weights the *belief* on the non-uniformity from the perspective of the target pixel. Likewise, $\beta$ in Eq. (1b) is defined as ${ }\overline{h}_{min}^{t} \left( {x,y} \right)/{ }\overline{h}_{T}^{t} \left( {x,y} \right)$. Clearly, 0 $\le \alpha ,{ }\beta \le 1$. ${\mathcal{H}}_{max}^{Bn}$ given in Eq. (3) represents the upper bound of entropy defined by ${\varvec{M}}^{t} ,{\text{ and}}$ ${\mathcal{H}}^{t} \left( {x,y} \right){ }$ equals ${\mathcal{H}}_{max}^{Bn} { }$ only when all bins are equal in height. At this stage, one should be able to see that due to the NMS-like random assignment in preparing ${\varvec{\theta}}_{\tau} {\text{ and }}\overline{{{ }{\varvec{\theta}}}}_{\tau}^{} ,{ }$ the gradient histogram of pixels covered by $M^{t}$ in a sense serve to simulate the local distribution of $\Omega$ or $\overline{\Omega }$. Later we will elaborate why the convergent likelihood $p^{c} \left( {x,y|\Omega } \right)$ or simply ${ }p^{c}$ can plausibly represent the estimated probability of observing the target pixel $\left( {x,y} \right)$ given the condition that it is from the gestalt class $\Omega$. That is, the larger ${ }p^{t}$ is, the more likely the pixel is located on a gestalt contour. The goal is to maximize ${ }p^{t}$ by alternatively updating the unknown parameters $L^t$ and $W^{t}$. That is, although $p^{t}$ is not directly observable, it can be inferred from $h_{i}^{t} \left( {x,y} \right)$ using Eqs. (1a) and (2a).

*Step(c):* This step and *Step(d)* form the M-phase in the EM algorithm. Let  $r=\sqrt {B_{n}^{} }$, $L$ and $W$ are updated as:

4 $$W^{t} = \left\{ {\begin{array}{*{20}l} {\left[ {r*(1 - p^{t} )} \right],} \hfill & {if\;p^{t} { \geqq }p^{{t - 1}} \;and\;\left[ {r*(1 - p^{t} )} \right] > 1} \hfill \\ {\left[ {r*(1 - p^{t} )} \right],} \hfill & {if\;p^{t} < p^{{t - 1}} \;and\;\left[ {r*(1 - p^{t} )} \right] > 1} \hfill \\ {1,} \hfill & {otherwise} \hfill \\ \end{array} } \right.$$

5 $$L_{}^{t} = \frac{{B_{n}^{} { }}}{{W_{}^{t} }}$$

*Step(d):* If $W^{t} = W^{t - 1}$, convergence reached, stop and output the convergent likelihoods; else, go to *Step(e)*.

*Step(e):* Deform and rotate the mask ${\varvec{M}}^{t}$, go to *Step(a)*.

Iterate *Step(a)* through *Step(e)* until convergence.

**Figure 5:**
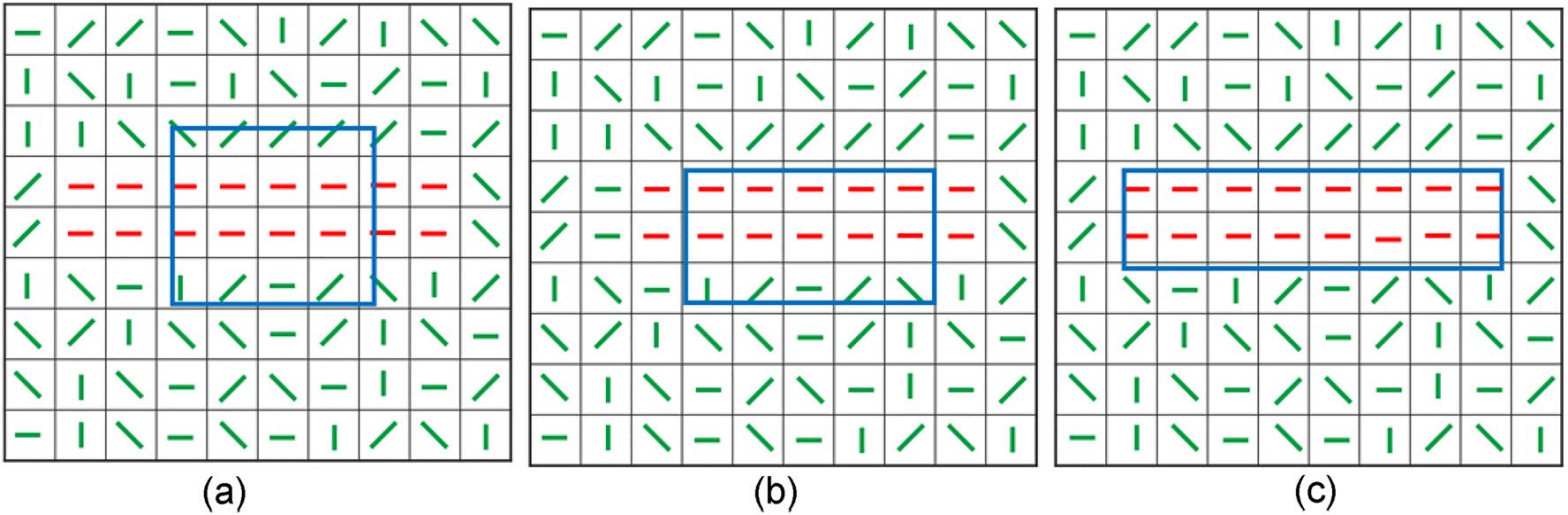
(**a**) $$L = 4, W = 4$$. (**b**) $$L = 5, W = 3.2$$(discretized to 3). (**c**) $$L = 8, W = 2$$.

**Figure 6:**
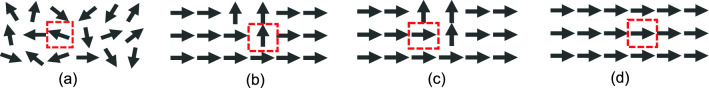
The likelihood value is different as local image regularities vary. (**a**) $p$ = 0, the target pixel cannot be a Gestalt edge. (**b**) $p$ = 0.17, a rather low likelihood, the target pixel is unlikely a Gestalt edge, could be a noise. (**c**) $p$ = 0.88, the target pixel is very likely a Gestalt edge. (d) $p$ = 1, perfect likelihood, the target pixel is undoubtedly a Gestalt edge.

The rationality for the update Eq. (4) is explained as follows. The floor function and the ceiling function are used to ensure to ensure the updated integer value of W fall within the desirable range $\left[1,\lceil r \rceil\right]$. Equation (4) is designed in such a way to ensure that when $p^{t} { \geqq }p^{t - 1} ,{ }$ which represents more pixels covered by the mask are directionally in line with the target pixel and hence possibly form a principal direction, the width *W* should become smaller. Thus, if $p^{t} { \geqq }p^{t - 1}$, then the new value of *W* should take on the floor integer, so as to make the mask elongate by Eq. (5), enabling the mask to further align with the principal direction. Conversely, when $p^{t} < p^{t - 1} ,{ }$ *W* should become larger. Iterating in this way, the mid-level feature of principal direction associated with a contour can be found upon convergence. Also, with $r = \sqrt {B_{n} }$ and $Bn = L\times W$ and the inequality criteria set forth by Eqs. (4) and (5), it is easy to prove that the lower and upper bounds of $W$ and $L$ are $\left(1, \lceil r\rceil\right)$ and $\left(\lfloor r\rfloor ,Bn\right)$, respectively. This updating process is schematically shown in Fig. 5, where an initial square ${\varvec{M}}^{t}$ deforms to explore the existence of a principal direction in the local region surrounding the target pixel (*x, y*). Figure 5a shows an initial ${\varvec{M}}^{t = 0}$ with $L = 4$, $W = 4$. After the *1*^*st*^ iteration, the sampling mask driven by the EM algorithm deforms (see Fig. 5b) in response to the more zero-degree gradients (red line fields). After the $2^{nd}$ iteration, as can be seen in Fig. 5c ${\varvec{M}}^{t}$ was further elongated ($L = 8$, $W = 2$) in response to the more pixels directionally in line with the target pixel covered by the updated mask in Fig. 5b. Through such a direction-aware sampling scheme, our method can automatically find out proximal pixels that share identical or similar gradient orientations with the target pixel. As will be proved later, our method ensures the maximal likelihood upon convergence. In the context of Bayesian inference, the sampling mask deforms iteratively to enhance the belief in the likelihood, it will eventually cover neighboring pixels from which the maximum likelihood estimation of $p^{t}$ can be obtained. During the iteration, the mask may rotate, and its shape will become narrower (wider) in response to a greater (smaller) likelihood. Such deformation not only facilitates pixels having the same or similar orientations to be covered by ${\varvec{M}}^{t}$, but also help discriminate a target pixel from pixels that have dissimilar gradient orientations, thus helping suppress noise and textures. This can be seen by comparing Fig. 6b,c, wherein the two target pixels (red box) in them are located in the same local area and hence has the same Shannon statistic (non-uniformity). But the small value of $\alpha$ in Fig. 6b guarantees a small likelihood, whereas the large value of $\alpha$ in Fig. 6c results in a high likelihood. This is why our method can suppress noise and texture effectively. Also, since both parameters $\alpha$ and non-uniformity are solely derived from angle data, the likelihood $p$ is irrelevant to the gradient magnitude, thus enabling our method to effectively detect low-contrast contours as well.

### The objective function and stability analysis

Considering that MSE (Mean Squared Error) is always convex on its input and parameters by itself, in order to prove the stability of the iterative sampling scheme, we can conveniently prescribe an objective (Lyapunov) function $E^{t} \left( {x,y} \right)$ in the context of MSE between the new and old values of $W$ as in Eq. (6)

6 $$E^{t} \left( {x,y} \right) = \frac{1}{{n\left\{ {{\text{CEP}}} \right\}}}\mathop \sum \limits_{{\left( {{\text{x}},{\text{y}}} \right) \in {\text{CEP}}}} \left[ {W_{}^{t} - r{*}(1 - p^{t} \left( {x,y} \right))} \right]^{2}$$

And the stability analysis is cast into a convex optimization problem, the derivative of ${ }E^{t} \left( {x,y} \right)$ w.r.t the target pixel $p^{t} \left( {x,y} \right)$ is calculated as in Eq. (7)

7 $$\Delta E^{t} \left( {x,y} \right) = 2r \times \left[ {W_{}^{t} - r{*}(1 - p^{t} \left( {x,y} \right)} \right]\Delta p^{t} \left( {x,y} \right)$$

where $\Delta p^{t} \left( {x,y} \right) = p\left( {x,y} \right)^{t} - p\left( {x,y} \right)^{t - 1}$. According to Eq. (4),

if $\Delta p^{t} \left( {x,y} \right){ \geqq }0,{\text{ then }}W_{}^{t}$ will take on the floor value of $r{*}(1 - p^{t} \left( {x,y} \right))$, ensuring the term $\left[ {W_{}^{t} - r{*}(1 - p^{t} \left( {x,y} \right))} \right] < 0$, thus $\Delta E^{t} \left( {x,y} \right) \le 0.$

If $\Delta p^{t} \left( {x,y} \right) < 0,$ ${\text{then }}W_{}^{t}$ will take on the ceiling value of $r{*(1-p}^{t}(x,y)),$ ensuring the term $\left[ {W_{}^{t} - r{*}(1 - p^{t} \left( {x,y} \right))} \right] > 0$, thus $\Delta E^{t} \left( {x,y} \right) < 0.$

In short, $\Delta E^{t} \left( {x,y} \right)$ is never positive. Thus, iteratively updating $L_{}^{t} {\text{ and}}$ $W_{}^{t}$ with Eqs. (4) and (5) guarantees at least a local minimum convergence of $E^{t}$. To see the rationale here, we denote the aspect ratio  $\wedge^{t} =L_{}^{t}$*/*$W_{}^{t}$ and assume $E^{t} \left( {x,y} \right) = 0,$ i.e., $W_{}^{t} = r \times (1 - p^{t} \left( {x,y} \right)).{ }$ In an extreme case, if $p^{t} \left( {x,y} \right) \to 1,$ then $W_{}^{t} \to 0$, meaning the sampling mask has the highest aspect ratio, strongly indicating the existence of a principal direction. Conversely, if $p^{t} \left( {x,y} \right) \to 0$, then $W_{}^{t} \to r$ and $\wedge^{t} \to 1$, meaning a rectangular sampling mask and hence no existence of a principal direction. Finally, since $r$ is a constant and $r^{2} = L_{}^{t} \times W_{}^{t}$, when ${ }L_{}^{t} { }$ is updated to become more in line with an existing principal direction, the value of $p^{t} \left( {x,y} \right)$ also gets larger. In short, through alternatively updating the parameters $L_{}^{t} { }$ or $W_{}^{t} ,$ the EM algorithm guarantees to find a principal direction that, if it does exist, provides sufficient evidence of a target edge being located on an object contour.

### Qualitative analysis on the likelihood

For brevity, we only use (1a) as an illustrative example to explain the rationale of Eqs. (1a) and (1b). We start by noting that the entropy ${\mathcal{H}}^{t} \left( {x,y} \right)$ calculated using Eq. (2) inversely stands for the orientation resemblance between the pixels covered by ${ }{\varvec{M}}^{t}$. Using $h^{t} \left( {x,y} \right)$ to compute ${\mathcal{H}}^{t} \left( {x,y} \right)$ allows to conveniently evaluate the orientation resemblance within ${\varvec{M}}^{t}$. Without the deformation scheme, ${\mathcal{H}}^{t}$ would merely account for image entropy surrounding the target pixel. Namely, a larger ${\mathcal{H}}^{t} \left( {x,y} \right)$ represents a more uniform distribution in ${ }h^{t} \left( {x,y} \right)$, hence it will be less likely to see a principal direction. Because the existence of principal direction is a necessary condition for $\Omega$ to be the true class for the target pixel $\left( {x,y} \right)$, it seems quite reasonable to define the likelihood, or class-conditional probability, for the class $\Omega$ w.r.t the target pixel $\left( {x,y} \right)$ as

8 $$p^{t} \left( {x,y|\overline{\Omega }} \right) = 1 - \frac{{{\mathcal{H}}^{t} \left( {x,y} \right)}}{{{\mathcal{H}}_{max}^{Bn} }}$$

However, human visual perception is quite a complicated task, using ${\mathcal{H}}^{t} \left( {x,y} \right)$ alone is inadequate to accurately measure the likelihood for the target pixel. This can be seen by comparing Fig. 6b,c, where both have the same value of ${\mathcal{H}}^{t} \left( {x,y} \right)$, but clearly the target pixel in Fig. 6c should be more likely a gestalt edge. Considering this, a bias term ${ \mathcal{H}}_{bias}^{t} \left( {x,y} \right)$ is included as a compensating factor. We rewrite $p^{t}$ of Eq. (8) as

9 $$p^{t} = 1 - \left\{ {\frac{{{\mathcal{H}}_{Ef}^{t} \left( {x,y} \right)}}{{{\mathcal{H}}_{max}^{Bn} }}} \right\} = 1 - \left\{ {\frac{{{\mathcal{H}}\left( {x,y} \right) + {\mathcal{H}}_{bias}^{t} \left( {x,y} \right)}}{{{\mathcal{H}}_{max}^{Bn} }}} \right\},$$

where the effective entropy ${\mathcal{H}}_{Ef}^{t} \left( {x,y} \right)$ = ${\mathcal{H}}^{t} \left( {x,y} \right) + {\mathcal{H}}_{bias}^{t} \left( {x,y} \right)$. According to Hypothesis-2, orientation similarities provide the cue for forming smooth contours, that is why we use the parameter $\alpha = h_{T}^{t} \left( {x,y} \right)/h_{max}^{t} \left( {x,y} \right){ }$ to specifically take account of the local bin strength of the target pixel relative to the highest bin (i.e., principal direction). Given the non-uniformity $\left[ {{\mathcal{H}}_{max}^{Bn} - {\mathcal{H}}\left( {x,y} \right)} \right]$, the bias term is prescribed as follow

10 $${ \mathcal{H}}_{bias}^{t} \left( {x,y} \right) = \left( {1 - \alpha } \right){*}\left[ {{\mathcal{H}}_{max}^{Bn} - {\mathcal{H}}^{t} \left( {x,y} \right)} \right]{ }$$

With such a design, given the same value of $\left[ {{\mathcal{H}}_{max}^{Bn} - {\mathcal{H}}^{t} \left( {x,y} \right)} \right],$ a smaller $\alpha$ tends to reduce the *belief* for the target pixel of Fig. 6b by adding more bias prescribed in Eq. (10). Conversely, a greater $\alpha$ tends to keep the more original entropy ${\mathcal{H}}^{t} \left( {x,y} \right){ }$ intact by allowing less bias added to the receptive field in Fig. 6c. By plugging Eq. (10) into Eq. (9), we readily obtain Eq. (1a).

Finally, it is interesting to note: (i) for one extreme case shown in Fig. 6a where ${ }{\mathcal{H}}^{t} = {\mathcal{H}}_{max}^{Bn} ,{\text{ so }}{ \mathcal{H}}_{bias}^{t} = 0$, according to Eq. (8), $p^{t} = 0$; (ii) for another extreme case shown in Fig. 6d, because $\alpha = 1,{ }{\mathcal{H}}_{bias}^{t}$ = 0, again, Eq. (9) is reduced to Eq. (8), due to ${ \mathcal{H}}^{t} = 0$ when $p^{t} = 1$ by Eq. (1a), a smaller ${\mathcal{H}}^{t}$ represents a better chance to see a principal direction. However, the non-uniformity $\left[ {1 - {\mathcal{H}}^{t} \left( {x,y} \right)/{\mathcal{H}}_{max}^{Bn} } \right]{ }$ statistically assess how centralized the gradient orientations are distributed over the sampled region, that is, it evaluates the chance of observing a principal direction formed by proximal pixels. To comply with both the laws of proximity and similarity, a complete definition for the likelihood should also consider the local information about how similar the target pixel is to the principal direction. This is exactly the role the belief parameter $\alpha$ plays in Eq. (1a). Given the fact that $\alpha$ specifically considers the gradient similarity between the target pixel and the principal direction, multiplying $\alpha$ by the non-uniformity value would plausibly yield a weighted belief on how likely the class $\Omega { }$ is truly gestalt. Because 0 $\le {\mathcal{H}}^{t} \left( {x,y} \right)/{\mathcal{H}}_{max}^{Bn} \le 1{ }$ and $0 \le \alpha \le 1$, ${ }p^{t}$ always falls in the range of [0,1]. If $p\left( {x,y|\Omega } \right){ }$ is large, it means the class $\Omega$ is more “likely” truly a gestalt class, and the probability of the target pixel being located on a gestalt contour should be high. Thus, parameter ${ }p^{t}$ serves as a probabilistic measurement of how likely the target pixel $\left( {x,y} \right) \in \Omega$. Likewise, it can be easily shown that $0 \le \overline{p}^{t} \le 1$.

Figure 6b shows a converged mask centered at a target pixel (enclosed by a square) with a gradient orientation represented by the symbol (↑). Among the total 18 pixels, only three of them are ↑, yielding $h_{T}^{t} \left( {x,y} \right) = 3$ and $h_{max}^{t} \left( {x,y} \right) = 15$, hence $\alpha = 0.2$. In contrast, Fig. 6c shows a converged mask centered at a target pixel with direction → , thus $h_{T}^{t} \left( {x,y} \right) = h_{max}^{t} \left( {x,y} \right)$ = 15, yielding the highest similarity $\alpha = 1$. Interestingly, Fig. 6b,c have the same histogram distribution, yet their converged masks look quite different. For the target pixel in Fig. 6c, $p =$ 0.885, which is a lot greater than that (0.178) in Fig. 6b. These computing results are consistent with human perception. They clearly show, by taking the target pixel as CRF and its surrounding pixels as NCRF respectively, that the parameter $\alpha$ can effectively suppress the impact of self-inhibition^10^, even given the same local distributions in the gradient orientations.

The significance of large $p$ values in Fig. 6c,d is that given the target pixel $\left( {x,y} \right) \in \Omega$, the chance of observing such orientation distributions should be high. Conversely, if a target pixel $\left( {x,y} \right) \in \Omega$, then it is very unlikely to observe orientation distributions as shown in Fig. 6a,b. Therefore, based on the above simple calculations, a mask deformed according to Eqs. (4) and (5) indeed is effective for evaluating the likelihood of observing a gestalt pixel. Although the target pixel in Fig. 6b satisfies the proximity law, i.e., the tendency to group pixels (covered by the same converged mask) into a meaningful identity, its small ${{ \alpha }}$ indicates the target pixel is rather dissimilar to the principal direction. Contrasting to Fig. 6b, the target pixel in Fig. 6c satisfies requirements of both proximity and similarity, and it has a large $p$ value, in response, the mask deformed into a more elongated shape than that in Fig. 6b. In short, comparing Fig. 6b with Fig. 6c justifies our claim that the direction-aware mask can exploit both local similarity (via $\alpha$) and spatial proximity (via the non-uniformity).

### Pixel classification using Bayes rule

In this work, the binary classification predictive problem is framed as a conditional probability model. Because an object contour normally contains a set of edges having high gradient magnitude, ${\varvec{g}}^{N}$ can be taken as the prior probability distribution for $\Omega$. With the convergent likelihood for the target pixel $\left( {x,y} \right)$ being located on a perceptual contour, $p^{c} \left( {x,y|\Omega } \right)$ is the probability of observing the event ${ }h^{c} \left( {x,y} \right)$ given the condition that the target pixel $\left( {x,y} \right) \in \Omega$. Also, $\overline{p}^{c} {\text{ is now }}$ formally written as $\overline{p}^{c} \left( {x,y|\overline{\Omega }} \right),$ namely the likelihood for the target pixel $\left( {x,y} \right)$ being located on a non-perceptual contour. According to Bayes theorem, the posterior probabilities $p\left( {\Omega |x,y} \right){ }$ and $p\left( {\overline{\Omega }|x,y} \right)$ are approximated respectively as in Eq. (11a) and (11b), respectively.

11a $$p\left( {\Omega |x,y} \right) = \frac{{p^{c} \left( {x,y|\Omega } \right) \times g^{N} \left( {x,y} \right)}}{{p^{c} \left( {x,y|\Omega } \right) \times g^{L} \left( {x,y} \right) + \left[ {p^{c} \left( {x,y|\overline{\Omega }} \right) \times \left( {1 - g^{N} \left( {x,y} \right)} \right)} \right]}}$$

11b $$p\left( {\overline{\Omega }|x,y} \right)\frac{{p^{c} \left( {x,y|\overline{\Omega }} \right) \times \left( {1 - g^{N} \left( {x,y} \right)} \right)}}{{p^{c} \left( {x,y|\Omega } \right) \times g^{L} \left( {x,y} \right) + \left[ {p^{c} \left( {x,y|\overline{\Omega }} \right) \times \left( {1 - g^{N} \left( {x,y} \right)} \right)} \right]}}$$

The decision on whether the target edge pixel is on a gestalt edge can be made in various ways, e.g., if $p\left( {\Omega |x,y} \right) > p\left( {\overline{\Omega }|x,y} \right)$, the target pixel is a gestalt edge. Here, we use the following rule: if $p\left( {\Omega |x,y} \right){ \geqq }k$, $0.4 \le k \le 0.9$, then the target pixel is classified as a gestalt edge. Moreover, we can mark any pixels ($x^{\prime},y^{\prime}$) covered by the converged mask as gestalt edges, too, provided they satisfy the condition $h_{}^{t} \left( {x^{\prime},y^{\prime}} \right) = h_{}^{t} \left( {x,y} \right)$, i.e., the two points ($x^{\prime},y^{\prime}$) and ($x,y$) are aligned on a line or curve, resulting in good agreement with the gestalt law of continuity, As such, a certain portion of edge pixels in CEP may be simply skipped by the EM algorithm due to such automatic labeling. In this context, the principal direction $h_{max}^{t} { }$ acts as a cue for instantly grouping proximal pixels into the same contour. Empirical test on datasets of RUG^10^ and BSDS^8^ shows that roughly 15% of the total pixels in CEP fall into this category. This time-saving property makes our method different from^10^ where the tangent vector for each pixel needs to be *separately* calculated. Note that the parameter $k$ corresponds to the confidence threshold used in plotting the ROC curve for evaluating the performance of a general classifier. After all pixels in CEP have been classified, they together form the final contours.

## Results

Extensive experiments were conducted, qualitative and quantitative characterizations thereof are provided to verify the effectiveness of our method. Unless otherwise specified, ($H_{t} { },k$) = (0.2, 0.5). $B_n$  is set to one-fourth the sampling mask. All experiments were coded with Matlab and executed by Intel i7 8700 CPU and 32 GB RAM. Although the proposed deformable mask operates analogously to the convolution operation employed in Canny detector in that sense that our mask also processes pixels of CEP one by one, it does not scan pixels sequentially all over the image plane due to the random dynamics of EM-algorithm. Besides, the total number of pixels of which is not fixed, so the convolution theorem unfortunately cannot apply here to reduce the computation complexity from $O(n^2)$ to $n*log(O(n))$, where n is the 2D image size.

### Preserving smoothly varying contours

**Figure 7:**
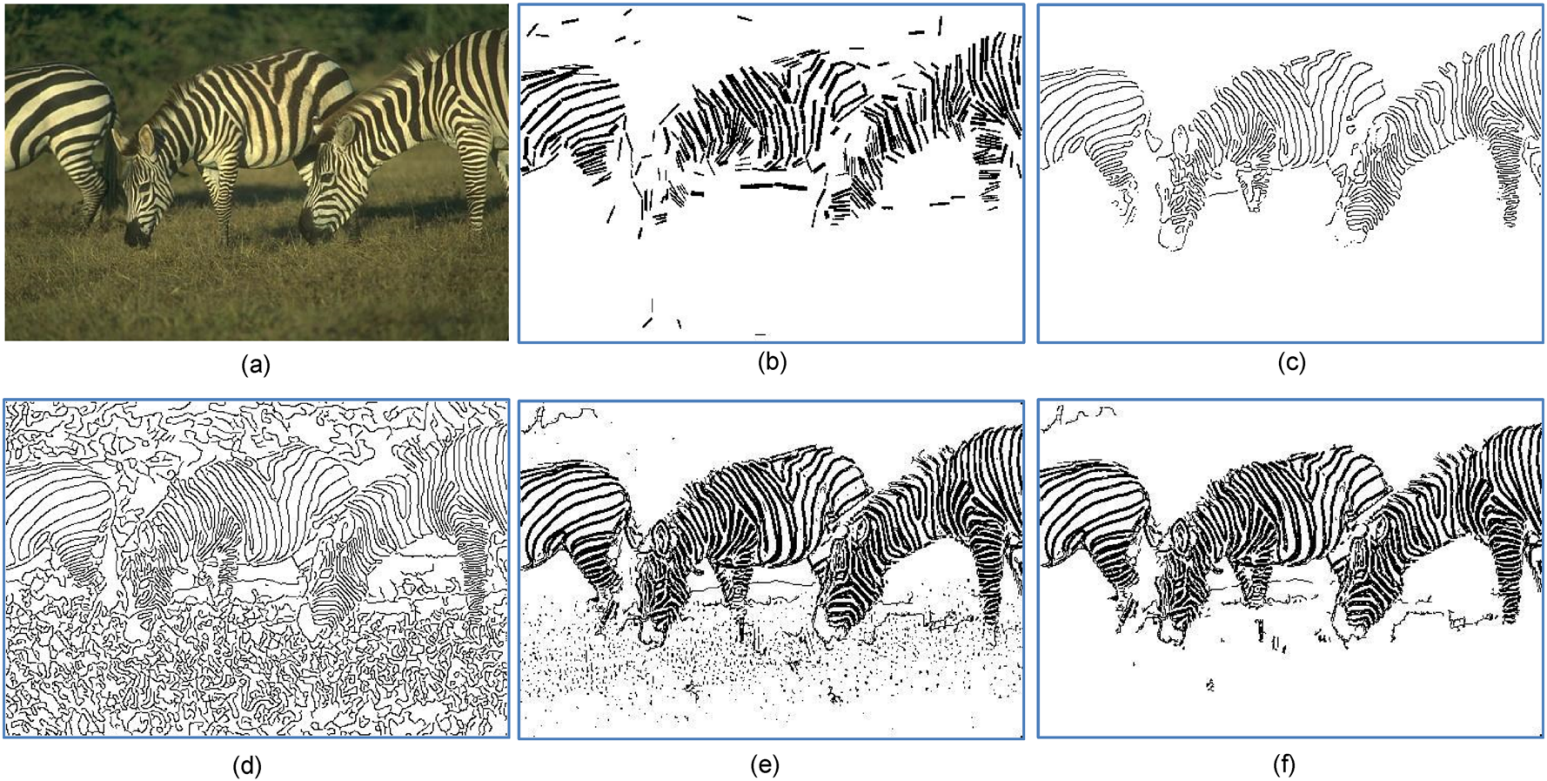
(**a**) A complex nature image. (**b**) Contours extracted by LSD. (**c**) Contours extracted by our method using Canny’s CEP. (**d**) CEP generated by Canny detector ($$H_{t} = 0.2)$$. (**e**) CEP generated by (Gabor + Canny). (**f**) Contours by our method using CEP of (**e**).

**Figure 8:**
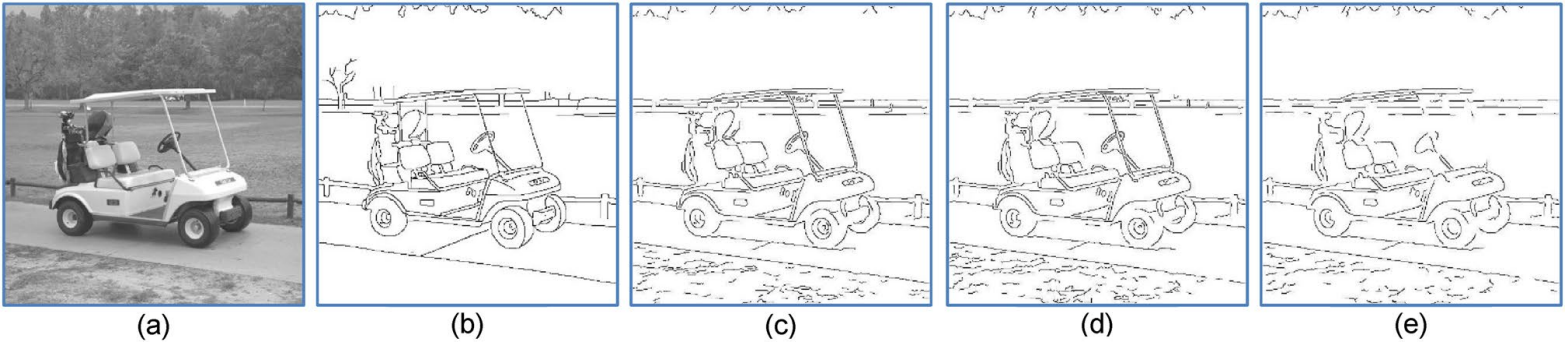
(**a**) Input image. (**b**) Ground Truth (**c**) Gaussian prefilter, (MQ, EQ, FoM) = (0.87, 0.84, 0.80). (**d**) Adaptive gaussian prefilter as prefilter, (MQ, EQ, FoM) = 0.86, 0.82, 0.78). (**e**) Median prefilter, (MQ, EQ, FoM) = (0.85, 0.85, 0.65).

Using Fig. 7a as the test image, Fig. 7b,c show detection results of LSD^22^ and ours, respectively. Clearly, our method can better preserve smoothly varying or curved contours. the method produced many straight and disconnected stripes contours on the Zebra’s back. Note our method is highly flexible in the sense that it is not limited to the use of Canny as the CEP generator. It is interesting to see how our method performs when using other CEP generators. Fig. 7f is the detection result from using the combined outputs of Canny and Gabor wavelet, the two images of which are shown in Fig. 7d,e for comparison. Clearly, much more smoothly varying(curved) contours can be preserved than those by any of Fig. 7b–e. To see the effect of prefilters on the performance of our method, Fig. 8c through Fig. 8e pictorially show the detection results of using Gaussian filter^38^, adaptive Gaussian filter^39^, and median filter as the prefilter, respectively. By comparing with the ground truth, we see that the Gaussian filter has the best qualitative performance, especially in preserving the true edges in many parts of the golf cart, such as tires, steering wheel, and roof support poles. However, unlike in Fig. 7, it is difficult to give a quantitative and objective judgment on which detection result is the best in terms of the entire picture. Thus, we need more reliable metrics to quantitatively measure the quality of detection results, i.e., quantifying the (dis)similarity between detected pixels (DP) and ground truth (GT).

### Performance metrics

Three widely used metrics are explained as follows. Firstly, the Pratt’s FoM (figure of merit) is defined as

$${ }\frac{1}{{max\left( {n\left\{ {{\text{DP}}} \right\};n\left\{ {{\text{GT}}} \right\}} \right)}}\mathop \sum \limits_{{\left( {{\text{x}},{\text{y}}} \right) \in {\text{DP}}.}} \frac{1}{{1 + \left[ {\frac{{{ }d\left( {{\text{x}},{\text{y}}} \right)}}{{d_{0} }}} \right]^{2} }},$$

where $d\left( {x,y} \right)$ represents a distance transform measure, and the scale parameter *d*_*0*_ controls the sensitivity of FoM to differences between GT and DP. Although FoM does not require exact spatial matching between the detected pixels (DP) and those of GT, it provides no information about the origin of the dissimilarity: false positives (FP) and false negatives (FN). To solve this problem, another approach^41^ is to use EQ (edge quality) and MQ (map quality), where ${\text{EQ}} = n\left\{ {{\text{GT}} \cap {\text{DP}}} \right\}/{ }n\left\{ {{\text{DP}}} \right\}$ and ${\text{MQ}} = n\left\{ {{\text{GT}} \cap {\text{DP}}} \right\}/\left\{ {n\left\{ {{\text{GT}}} \right\} + n\left\{ {{\text{FP}}} \right\}} \right\}$. Thus, EQ and MQ measure the fraction of true positives ${\text{TP}} = {\text{GT}} \cap {\text{DP}}$ with respect to the number of all detected pixels and the number of $\left\{ {{\text{GT}} + {\text{FP}}} \right\}$, respectively. EQ corresponds to the *Precision* rate. Also, if MQ is slightly modified into

12 $$MQ = \frac{{n\left\{ {TP} \right\}}}{{n\left\{ {TP} \right\} + 0.5 \times \left( {n\left\{ {FP} \right\} + n\left\{ {FN} \right\}} \right)}}$$

then Eq. (12) become the well-known F1-score that takes both FP and FN into account in evaluating classifiers. Either way, because the missed pixels FN are also counted, MQ is used to amend the drawback of EQ in measuring the quality of edges produced. To see this, assume that the detected pixels DP only comprises a very small number of both wrong and correct edges, EQ will measure a false ratio of one. However, due to a possible displacement error $\delta$ of the hand-sketch contours in GT with respect to their exact positions in the input image, the set TP can be empty even in the total absence of false positive and false negative. Therefore, in practice, one would simply replace TP with pixels of DP as long as whose distance from GT is no greater than 5 pixels, i.e., the scale parameter $d_{0} =$ 1, 2,…5. As in FoM, the parameter $d_{0}$ controls the sensitivity of EQ and MQ to the difference between GT and DP. A larger value of $d_{0}$ means a greater tolerance of difference between GT and DC. Comparing the values of (MQ, EQ, FoM) of Fig. 8b–d shows that using a Gaussian prefilter yields the best detection result. Except for rare cases (e.g., Figs. 13 and 14), our study showed that using a Gaussian prefilter generally produces better detection contours than otherwise.

### Test on open dataset

The Contour Image Database RUG^10^ comprising 40 grey images is ideally sufficient for evaluating human perceived contours. We compared our method with Grigorescu^10^, Papari^11^, Edge Drawing^12^, and Canny detector. All these five detectors exploit gradient information for various purposes. With $d_{0}$ = 1 (the most strict tolerance), Table 1 lists the averaged MQ, EQ, and FoM over the 40 images. Our method not only outperforms others but achieved the most balanced performance (i.e., least difference between MQ and EQ). Here, MQ corresponds to the ODS (rather than OIS.) F-score, with ODS (optimal dataset scale) employing fixed thresholds for all images in a dataset, whereas OIS (optimal image scale) selecting a set of optimal thresholds for each image. Analysis based on definitions of MQ and EQ provides some interesting observations. Firstly, if MQ $\approx$ EQ, then n(FP)$\approx$ n(FN), which is desired property, Because if MQ $\approx$ EQ, then it can be proved that when MQ≧0.5, n(TP)$\approx$ n(FP)$\approx$ n(FN), with n(DP)$=$ n(TP)$+$ n(FP), DP must be at least half coinciding with GT Thus, the conditions of MQ $\approx$ EQ and MQ > 0.5 ensure at least half of GT present in the output quality. We called this the Half-GT point. Secondly, the inferior EQ performance in Canny detector indicates it tends to produce much more falsely detected pixels (FP) than missed pixels (FN), i.e., FP >> FN Thirdly, the better MQ performance over the Canny detector reveals that our method indeed can screen off the falsely detected pixels (FP) generated by Canny detector.

**Table 1:** Performance comparisons of different methods, ${d}_{0}$=1.

	Ours	^8^	^12^	Canny	^11^	^10^
MQ	0.66	0.61	0.59	0.55	0.38	0.33
EQ	0.75	0.70	0.70	0.62	0.35	0.32
FoM	0.64	0.61	0.60	0.54	0.39	0.33

**Figure 9:**
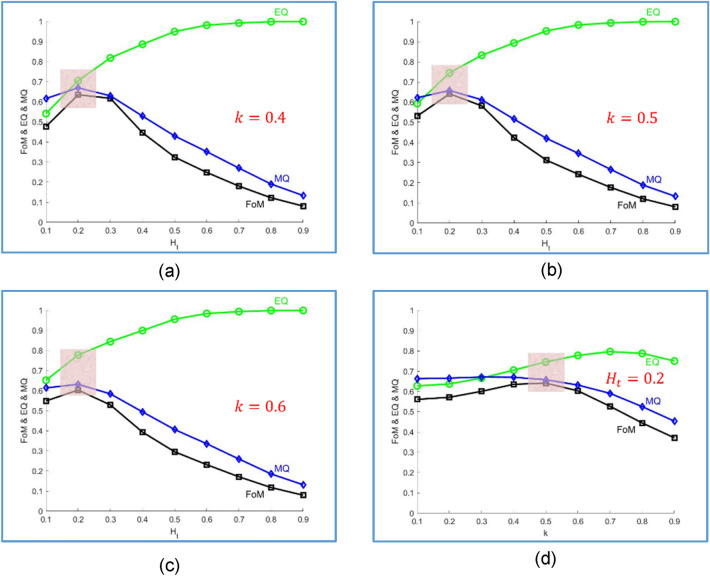
Performance indicators for (**a**)${ }k = 0.4$, (**b**)${ }k = 0.5$, (**c**)${ }k = 0.6$, (**d**)${ }H_{t} = 0.2$.

**Figure 10:**
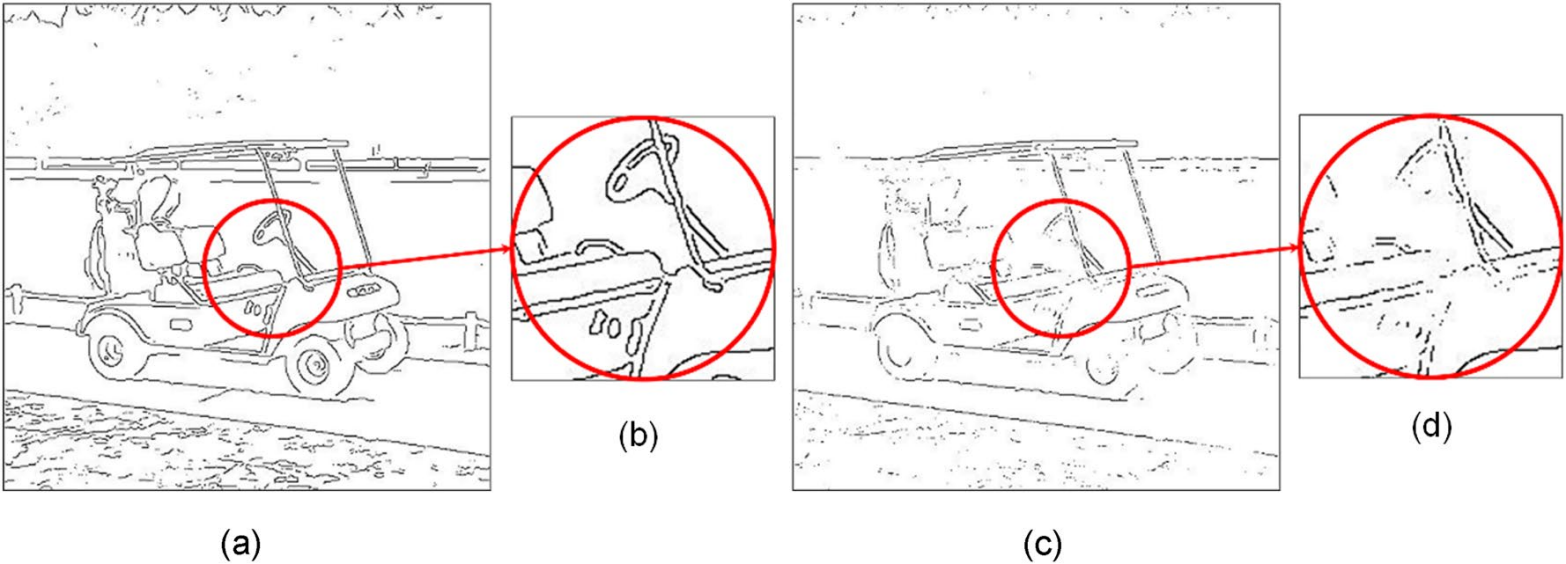
(**a**) $k =$ 0.5, (MQ, EQ, FoM) = ( 0.87, 0.84, 0.80). (**b**) Close-up for the encircled area in (**a**). (**c**) $k =$ 0.9, (MQ, EQ, FoM) = (0.55, 0.87, 0.45). (**d**) Close-up for the encircled area in (**c**).

By varying $H_{t}$ from 0.1 to 0.9, Fig. 9a–c show the averaged values of MQ, EQ, and FoM for $k = 0.5,{ }0.6,{ }0.7$, respectively. The lightly colored mask in Fig. 9b indicates a working zone, the center of which is the Half-GT point. Outside the colored mask, $\left| {{\text{MQ}} - {\text{EQ}}} \right|$ is greater than 0.2, it means either the set TP contains too few pixels or FP and FN are not neglectable, and the detection result should not be taken seriously. This rule-of-thumb is useful in judging the quality of detection results. For example, given $H_{t} > 0.3$ and $k > 0.5$, EQ increases rapidly with MQ and FoM dropping quickly. This is because if $H_{t}$ is getting larger, then more and more high-frequency pixels would be strongly smoothed out by Canny detector, leaving very few pixels in CEP and making the high EQ even less informative. With $H_{t}$ fixed at 0.2, Fig. 9d shows the averaged (MQ, EQ, FoM) for $k$ varying from 0.1 to 0.9. We see that the Half-GT point was reached at $k = 0.5$. This is significant, as the setting of $k = 0.5$ coincides with the optimality requirement in Bayes rule: namely, if $p^{c} \left( {\Omega x,y} \right){ \geqq }0.5$, the decision favors the class $\Omega$, and the error risk $p\left( {{\text{error}}|x,y} \right)$ is readily minimized as $min\left[ {p^{C} \left( {\Omega |x,y} \right),{ }p^{C} \left( {x,y|\overline{\Omega }} \right)} \right]$. With $H_{t}$ properly set, if a too large $k$ is used, then Eq. (11) will falsely classify excessive pixels as the class $\overline{\Omega }{ }$(i.e. FN increases rapidly), resulting in a large discrepancy between EQ and MQ. Figure 10 pictorially compares the results of applying our method to Fig. 8a using $k = 0.5$ and $k = 0.9$, respectively. Close-ups are provided, we see that $k = 0.9$ yields a high EQ and a much smaller MQ. To further characterize, we define a parameter of screening rate

13 $$sR = \frac{{n\left( {CEP - DT} \right)}}{{n\left( {CEP} \right)}}\%$$

**Figure 11:**
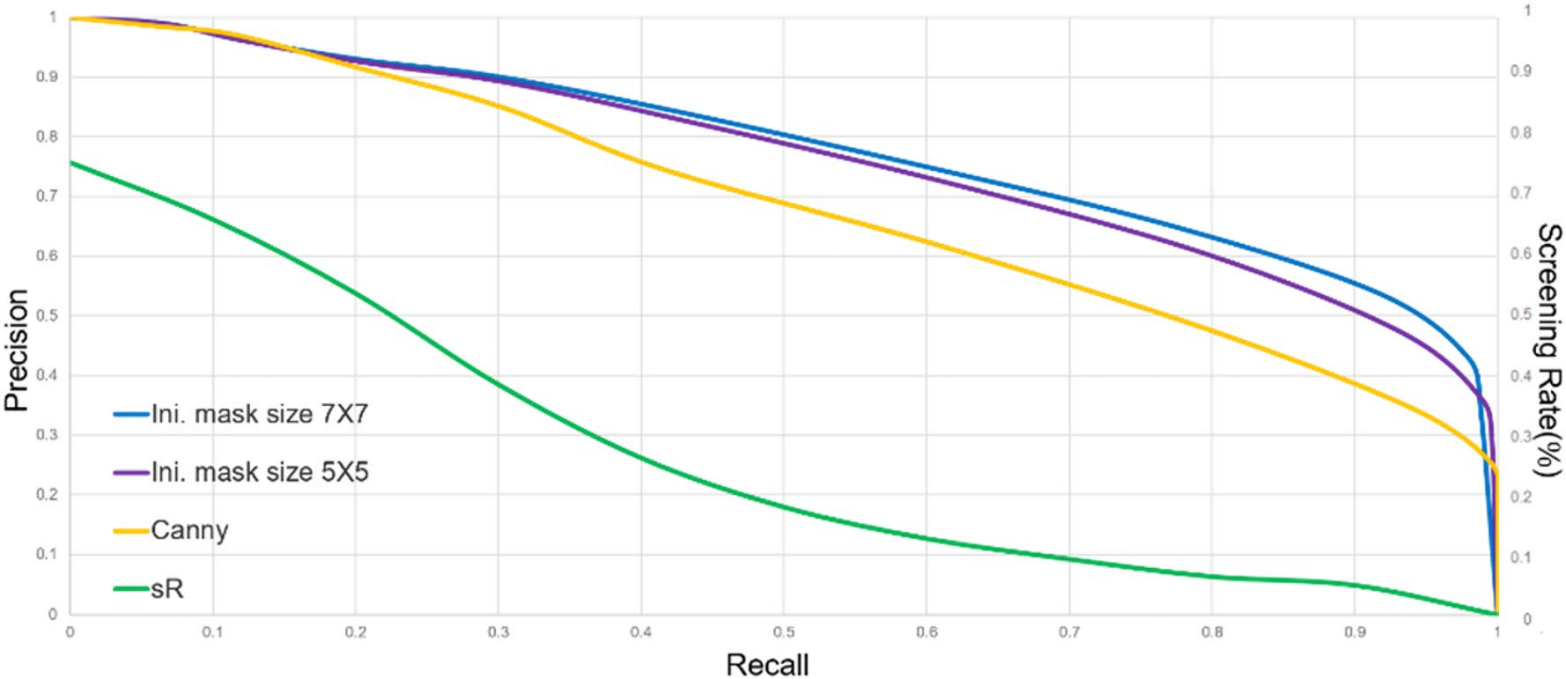
PR Curves of our method using initial mask size of 7*7 and 5*5. Yellow curve is for Canny detector.

*sR%* represents the percentage of CEP that were originally detected by Canny detector as TP, but identified as FP by our method and would be removed. Using RUG as the test dataset, Fig. 11 depicts the PR curves for our methods and Canny detector. Also shown is the corresponding *sR%* for each different combination of (*P, R*), with the average 31% in screening rate, justifying the effectiveness of our method. These results show that our method performs nearly the same when using initial mask size of 5*5 or 7*7.

In Table 2, the total number of edge pixels in CEP increases almost three times by decreasing $H_{t}$ from 0.3 to 0.1, and *sR%* goes up from 81 to 91%. Clearly, the total computation time required to process an input image varies with the value of $H_{t}$. A greater $H_{t}$ usually leads to fewer candidate pixels and less computation time, but it may adversely result in excessive FNs. Considering the recent rapid advancement in parallel computing hardware such as GPU, it is the computation time per pixel that really concerns us. Currently, using Matlab code executed in i7-8700 CPU, it takes our method 0.3 ms to process a pixel. In terms of computation load profile, 70% of computing resource was consumed by the iterative EM algorithm, and the rest 30% is taken up the prefilter, training data preparation, and Canny detection.

**Table 2:** Performance of using Median prefilter (RUG dataset), $k=0.5$.

	MQ	EQ	FoM	Average *sR*	*Card*(CEP)
Canny $(H_{t}$ = _0.3_)	0.61	0.83	0.58	23%	7116
Canny $(H_{t}$ = _0.2_)	0.66	0.75	0.64	39%	13,386
Canny $(H_{t}$ = _0.1_)	0.62	0.59	0.53	49%	26,160

### Robustness to CEP failure, noises, and interferences

**Figure 12:**
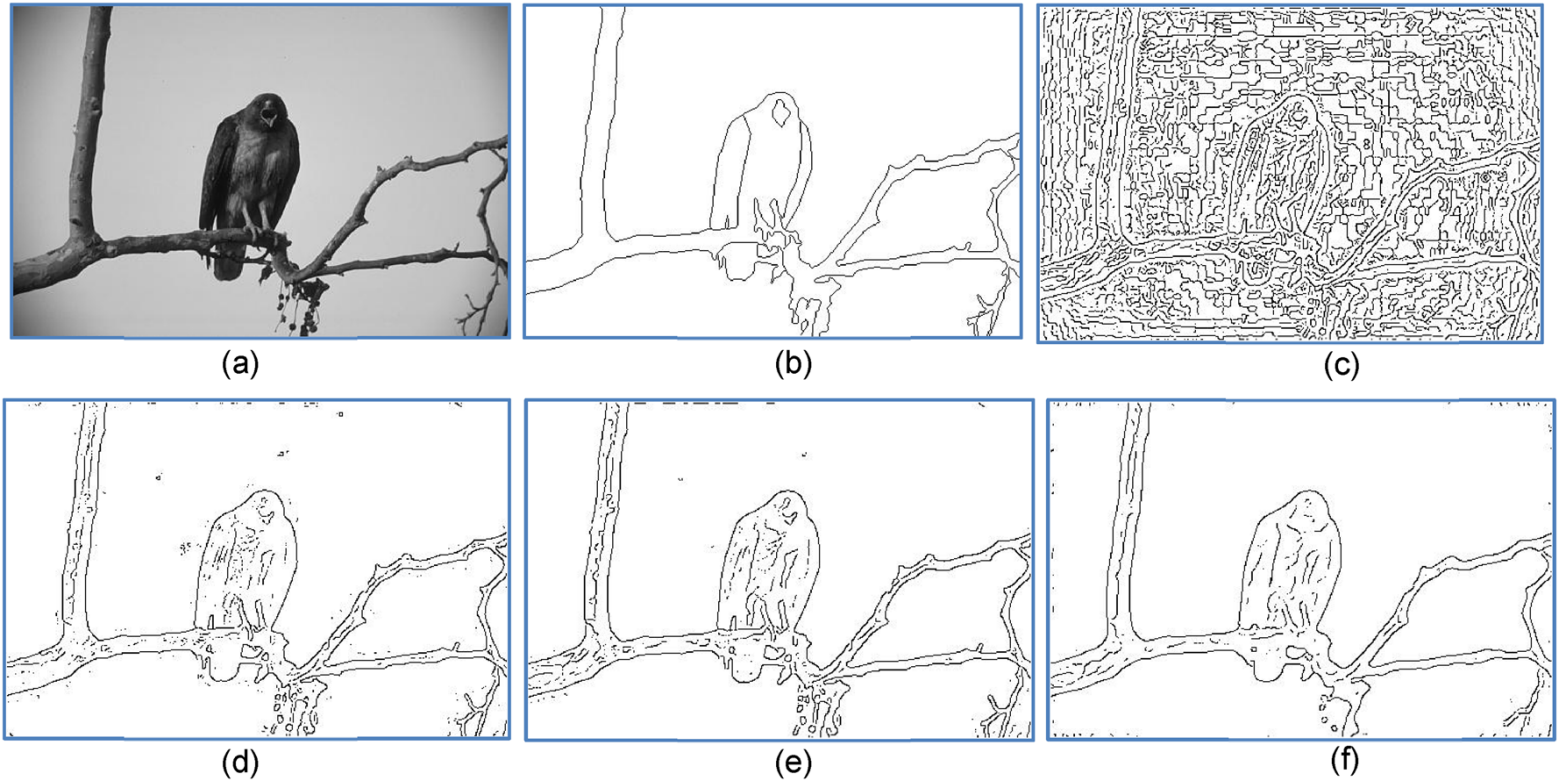
(**a**) Input image corrupted by Gaussian noise. (**b**) GT. (**c**) CEP generated by Canny detector ($$H_{t} = 0.01)$$, used as candidate pixels for (**d**–**f**), (**d**) Without prefilter, (MQ, EQ, FoM, sR) = (0.92, 0.85, 0.81, 74%). (**e**) Gaussian filter as prefilter, (MQ, EQ, FoM, sR) = ( 0.92, 0.85, 0.81, 74%). (**f**) Median filter as prefilter, (MQ, EQ, FoM, sR) = (0.72, 0.81, 0.81, 72%).

One may wonder how our method performs if the CEP generator fails. We intentionally set $H_{t} { }$ to nearly zero to simulate the poor thresholding in Canny detector. Using Fig. 12a as the test input image, Fig. 12b,c show the GT and excessive fragments produced by such a crippled CEP generator, respectively. Figure 12d–f show the results of using no prefilter, Gaussian prefilter, and median prefilter, respectively. They all achieved MQ $>$ 0.72 and *sR* $>$ 70%, indicating that our method has strong discriminating power, despite that the CEP contains nearly the Sobel output. They also justify that our method is robust to system faults such as a failure prefilter and/or non-functioning CEP generators.

**Figure 13:**
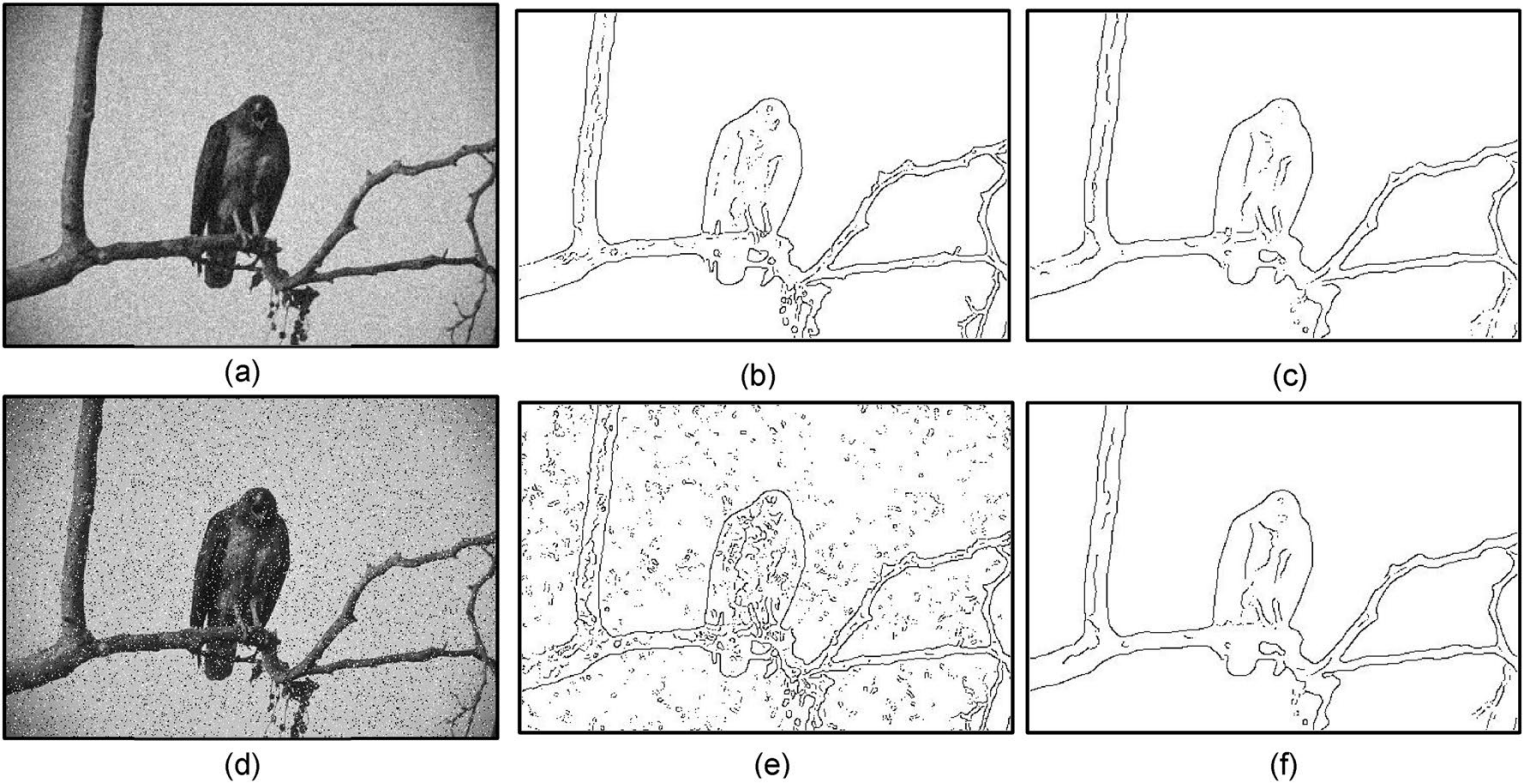
(**a**) Image corrupted by Gaussian noise(variance = 0.01). (**b**) Gaussian prefilter applied to (**a**), (MQ, EQ, FoM, sR) = (0.78, 0.92, 0.92, sR = 22%). (**c**) Median prefilter applied to (**a**), (MQ, EQ, FoM, sR) = (0.73, 0.91, 0.83, 16%). (**d**) image corrupted by impulsive noise (10%). (**e**) Gaussian prefilter applied to (**d**), (MQ, EQ, FoM, sR) = (0.64, 0.56, 0.56, 51%). (**f**) Median prefilter applied to (**d**), (MQ, EQ, FoM, sR) = (0.74, 0.90, 0.85, 10%).

Next, we added Gaussian and impulsive noises to the image of Fig. 12a and turned it into the test input images shown in Fig. 13a,d, respectively. For Fig. 13d, the results of using Gaussian prefilter and median prefilter are shown in Fig. 13b,c, respectively. For Fig. 13d, the corresponding detection results are shown in Fig. 13e,f, respectively. Based on three metrics, our method generally can preserve salient contours of objects in the presence of either gaussian or impulsive noises. Still, the result of Fig. 13b is better than that of Fig. 13c, and the result of Fig. 13e is inferior to that of Fig. 13f. This says, in order to obtain the best output contour, a right choice of the prefilter helps deal with a particular kind of noise. In contrast to Fig. 13a where the bird’s body contains lots of noises and textures, they can be effectively removed by our method, as shown in Fig. 13f.

**Figure 14:**
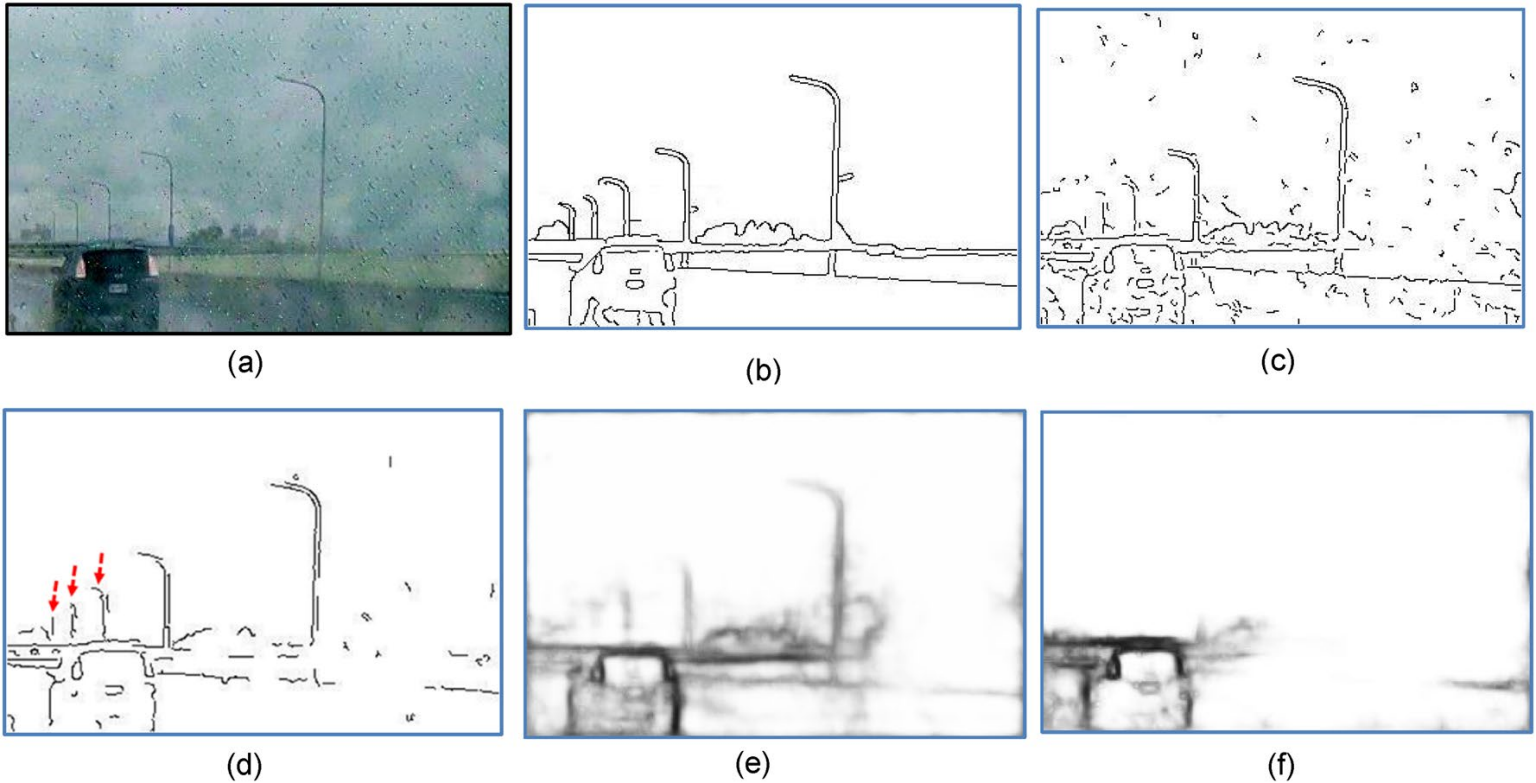
(**a**) Input image obscured by rains. (**b**) GT (contours perceived by human). (**c**) Gaussian prefilter, (MQ, EQ, FoM) = (0.67, 0.60, 0.60). (**d**) our method, (MQ, EQ, FoM) = (0.69, 0.89, 0.53). (**e**) RCF, (MQ, EQ, FoM) = (0.60, 0.50, 0.50). (**f**) HED, (MQ, EQ, FoM) = (0.49, 0.48, 0.48).

A more challenging test is shown in Fig. 14a, where a car is running under very bad weather. The optimal parameter setting ($k = 0.5,{ }H_{t} = 0.2$) was used. Figure 14c,d show the detection results when tested with Gaussian and median prefilters, respectively. As the raindrops are more like impulsive noises, the median prefilter yields a better detection result. Our method is robust as if the rains do not cause severe interferences, contours of the car, street light poles, and bridge were all well preserved. For comparison, the contour extraction results of RCF, HED are shown in Fig. 14e,f, respectively. As can be seen, there is a widening tendency in the width of edges generated by deep learning methods of HED and RCF, such thickening phenomenon will not happen in an edge-oriented approach like ours. In some applications such as medical image diagnostic where edge locating precision may be required to single pixel scale, such coarse contours output certainly is not desirable. Also, low-contrast objects, e.g. the $3^{rd}, 4^{th}, \text{ and } 5^{th}$ light poles were not preserved well by RCF, in comparison to ours (red arrows in Fig. 14d). The right half portion and light poles were completely missing in the output of HED. Also, in terms of (MQ, EQ, FoM), our method achieved (0.69, 0.89, 0.53), which outperforms RCF’s (0.60, 0.50, 0.50) and HED’s (0.49, 0.48, 0.48). With $d_{0}$ = 1, Table 3 compares the performance of HED, RCF, Canny detector, and our method using BSDS500^8^ which is a large benchmark dataset for evaluating object segmentation. Note that due to the nature of image-to-image transform, output results of HED and RCF are inherently grey images (not binary images as normally seen in other contour detectors), we have to convert them into binary images using Canny detector before calculating the values of (MQ, EQ, FoM).

**Table 3:** Performance Comparison using BSDS500 dataset, ${d}_{0}$=1.

	Ours $k = 0.5$	Canny $H_{t} = 0.2$	RCF	HED
MQ	0.76	0.57	0.53	0.62
EQ	0.69	0.44	0.61	0.59
FoM	0.61	0.38	0.53	0.57

## Discussions

Some observation highlights of the presented experimental results are summarized as follows. Our method requires no tedious parametric settings, a simple combination ($k$ = 0.5, $H_t$ = 0.2) generally yields quality contours with ${\text{MQ}} > 0.5$ and ${ }\left| {{\text{MQ}} - {\text{EQ}}} \right| < 0.2$. Also, we have shown the setting of $k = 0.5$ coincides with the optimality requirement in the Bayes rule. All these claims have been justified by the experimental results, showing our method outperforms other gradient-based detectors. Quantitative and qualitative characterizations on EQ/MQ/FoM indicators have helped devise criteria for performing objective judgment on the validity of pixelwise contour extraction results. Our method requires no tedious parametric settings, with *τ* being set to median or mean of elements in the quantized matrix ${\varvec{\theta}}$, a simple combination ($L_{t=0}$ = 7, $W_{t=0}$ = 7, $k$ = 0.5, $H_{t}$ = 0.2) generally yields quality contours with ${\text{MQ}} > 0.5$ and ${ }\left| {{\text{MQ}} - {\text{EQ}}} \right| < 0.2$. Also, we have shown the setting of $k = 0.5$ coincides with the optimality requirement in the Bayes rule.

The proposed bio-inspired method is robust even in case of non-functioning CEP generators and failures of prefilters, and it is resilient to noises and textures which can be suppressed by: (1) the prefilter, (2) the NMS imposed on the quantized orientation data in ${\varvec{\theta}}_{ \tau}^{} {\text{and }}\overline{{{ }{\varvec{\theta}}}}_{\tau}^{}$, or (3) the deformable mask that helps discriminate a target pixel from neighboring pixels that have dissimilar gradient orientations, which can be clearly seen by comparing Fig. 6b,c, wherein the two target pixels (red box) in them are located in the same local area and hence has the same non-uniformity. But after the modulation of the small value of $\alpha$ in Fig. 6b, the resulting likelihood is small, whereas the large value of $\alpha$ in Fig. 6c results in a high likelihood. That is why we call $\alpha$ as the belief degree on the principal direction. Figure 12f and Fig. 13f verify this superior ability in suppression noise and texture. In addition, from Eq. (1), we see both $\alpha$ and non-uniformity are solely derived from angle data, the likelihood $p$ is irrelevant to the gradient magnitude, thus enabling our method to effectively detect low-contrast contours as well. This also has been confirmed by the results in Fig. 14.

**Figure 15:**
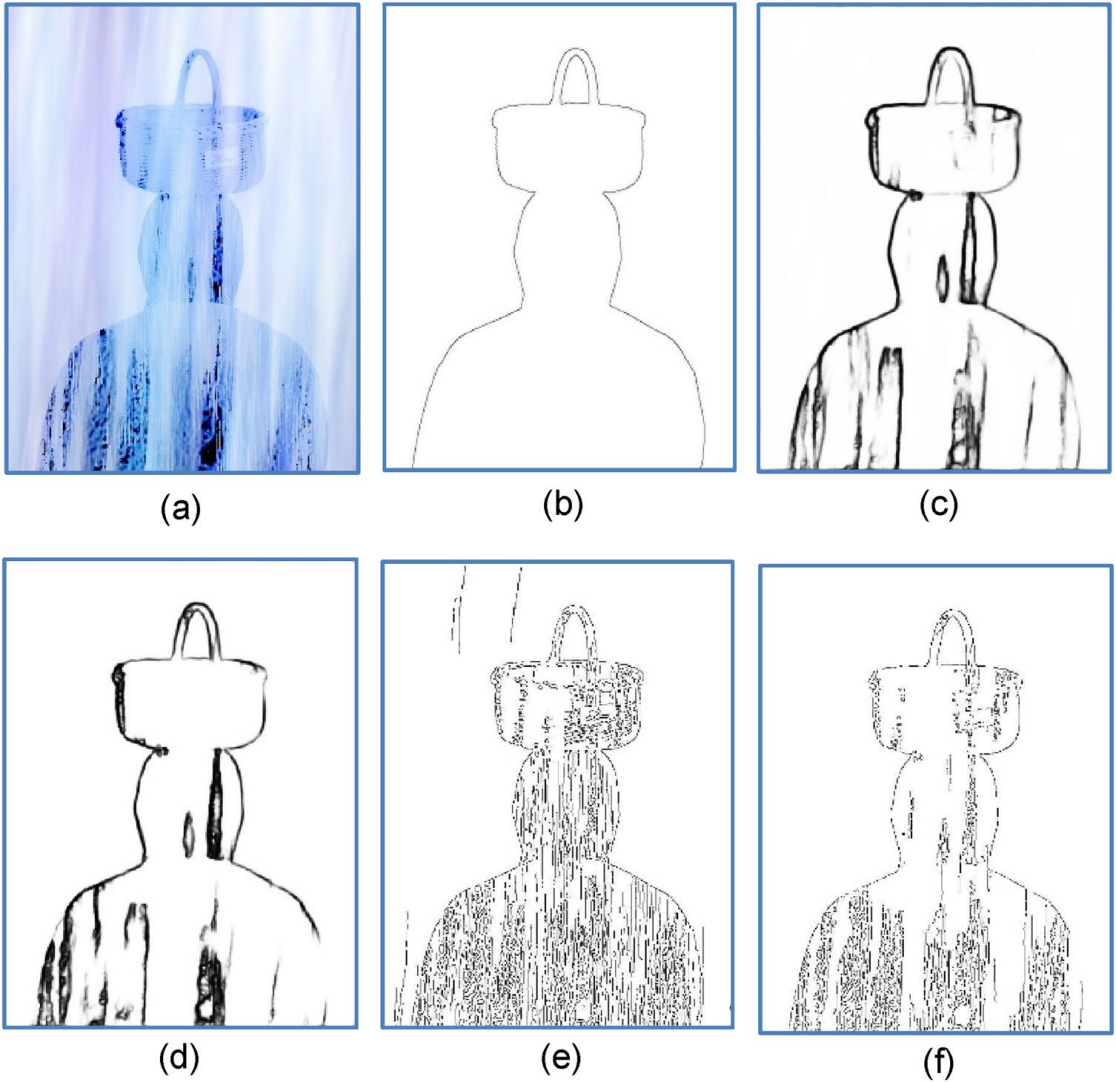
Failure case (**a**) Test image (**b**) GT(ground truth) (**c**) RCF, (MQ, EQ, FoM) = (0.09, 0.05, 0.18). (**d**) HED, (MQ, EQ, FoM) = (0.08, 0.04, 0.16). (**e**) Canny with $H_{t} = 0.1$, (MQ, EQ, FoM) = (0.05, 0.02, 0.06). (**f**) our method, (MQ, EQ, FoM) = (0.07, 0.04, 0.09).

**Figure 16:**
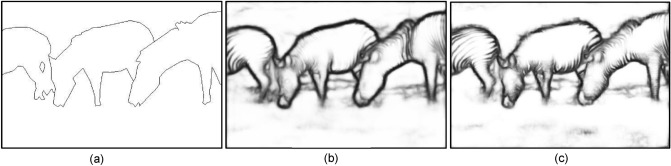
(**a**) GT form^8^. (**b**) RCF, (MQ, EQ, FoM) = (0.63, 0.46, 0.36). (**c**) HED, (MQ, EQ, FoM) = (0.65, 0.48, 0.38).

It is interesting to see what conditions under which the proposed method would fail to produce a gestalt object contour? We tested a special nature image of Fig. 15a comprising heavy interferences of rainfalls and an object of man with basket atop the head. Fig. 15b gives the gestalt object contour perceived by human eyes (GT). The results of RCF, HED, Canny detector, and our method are shown in Fig. 15c–f, respectively. All the four methods failed, to somewhat different degrees, in this test image. None of them can get rid of the extreme interferences. The heavy rainfalls, which are all over the image plane, contain gradient features that are too strong to be suppressed by our method. In the case of our method, the result in Fig. 15f came no surprise, and is due to the salient orientations inherent with the streamline-like rainfalls. But it is virtually impossible to explain why both the region-based models of HED and RCF erroneously preserved those rainfall textures inside the target object, as they were trained to learn the ground truth in Fig. 15b. To see the *labeling bias* problem, we also tested the zebra picture of Figs.7, 16b,c show the contour detection results of RCF and HED, respectively. The most distinctive difference between their resulting contours from ours in Fig. 7c or Fig. 7f are the strips components on the zebra’s body. Many strips were removed by RCF and HED, but nearly all of them were preserved by our method. As seen in Fig. 14e,f, performances of RCF and HED in terms of MQ, EQ, and FOM are not pretty due to the widening contours. Although the labeler of BSDS-500 draw the picture of Fig. 16a containing no strips as GT, we believe many people would disagree with that. In real life, human’s eyes will not let go those strips as they do to the grasses on the ground. Ironically, zebras evolved to have strips for blending into the environment to become stealthy to their predators.

Note that although our method in its present form is executed at pixel-level serially, that doesn’t mean the EM training process cannot be done in other ways. In fact, no data dependency is required in any of our algorithmic steps in Fig. 4. In this regard, it would be interesting to integrate our method with CNN paradigms such as HED^13^ or RCF^14^, so that features of pixel-level and higher levels can be learned more effectively and faster. This idea is inspired by the findings of^15^, who reported that features in the second convolution layer (Conv2) contribute the most, and the local edge information in low-level features and the object contour information in higher-level features are both necessary for achieving high performance in contour extraction tasks. Thus, by placing our converged likelihoods at Conv2 to act as a pre-trained probability map, the lengthy training time problem normally encountered in deep learning nets should be greatly alleviated.

Finally, in many vision-related applications, it is desired to have closed contours^42–44^. However, no state-of-the-art methods, including those mentioned in his study, can ensure highly (if not completely) closed bounding contours from a nature scene image. Thus, another future work can be directed to the key challenge in contour closure, i.e., connecting a set of fragmented contours into a cycle that separates an object from its background. In this regard, the output edge image ***P***, which is generated by applying the proposed method to CEP, can readily serve as a good start for the connection task. Because ***P*** is a set of gestalt edges, we can prepare a blank matrix ***C*** having the same size of ***P*** to store information of the principal direction and the shape of the converged mask for each pixel in CEP. Then, for each gestalt edge, retrieve its corresponding converged mask and check if there are any disconnected gestalt edges that share the same (or similar enough) information of principal direction and shape with the target edge, then they should form contiguous components of the same contour. In the case of Fig. 2b, where the red circle C-D represents a disconnected region (wherein F denotes an edge point falsely detected) between two subsets of edges forming the same contour, if the convergent masks of C and D gestalt edges overlap and share the same orientation in their principal direction, they should be connected. In addition, the more elongated their masks are, the more likely they belong to the same contour.

## Data Availability

The datasets generated during and/or analysed during the current study are available in the repository: RuG dataset: https://www.cs.rug.nl/~imaging/databases/contour_database/contour_database.html; BSDS500:https://www2.eecs.berkeley.edu/Research/Projects/CS/vision/grouping/resources.html.
